# On the utility of RNA sample pooling to optimize cost and statistical power in RNA sequencing experiments

**DOI:** 10.1186/s12864-020-6721-y

**Published:** 2020-04-19

**Authors:** Alemu Takele Assefa, Jo Vandesompele, Olivier Thas

**Affiliations:** 1 Department of Data Analysis and Mathematical Modeling, Ghent University, Ghent, 9000 Belgium; 2 Department of Biomolecular Medicine, Ghent University, Ghent, 9000 Belgium; 3 Cancer Research Institute Ghent, Ghent University, Ghent, Belgium; 4 Center for Medical Genetics, Ghent University, Ghent, Belgium; 5 National Institute for Applied Statistics Research Australia (NIASRA), University of Wollongong, Wollongong, Australia; 6 Data Science Institute, I-BioStat, Hasselt University, Hasselt, Belgium

**Keywords:** RNA sample pooling, RNA sequencing, Differential gene expression, Experimental design, Statistical power, Cost

## Abstract

**Background:**

In gene expression studies, RNA sample pooling is sometimes considered because of budget constraints or lack of sufficient input material. Using microarray technology, RNA sample pooling strategies have been reported to optimize both the cost of data generation as well as the statistical power for differential gene expression (DGE) analysis. For RNA sequencing, with its different quantitative output in terms of counts and tunable dynamic range, the adequacy and empirical validation of RNA sample pooling strategies have not yet been evaluated. In this study, we comprehensively assessed the utility of pooling strategies in RNA-seq experiments using empirical and simulated RNA-seq datasets.

**Result:**

The data generating model in pooled experiments is defined mathematically to evaluate the mean and variability of gene expression estimates. The model is further used to examine the trade-off between the statistical power of testing for DGE and the data generating costs. Empirical assessment of pooling strategies is done through analysis of RNA-seq datasets under various pooling and non-pooling experimental settings. Simulation study is also used to rank experimental scenarios with respect to the rate of false and true discoveries in DGE analysis. The results demonstrate that pooling strategies in RNA-seq studies can be both cost-effective and powerful when the number of pools, pool size and sequencing depth are optimally defined.

**Conclusion:**

For high within-group gene expression variability, small RNA sample pools are effective to reduce the variability and compensate for the loss of the number of replicates. Unlike the typical cost-saving strategies, such as reducing sequencing depth or number of RNA samples (replicates), an adequate pooling strategy is effective in maintaining the power of testing DGE for genes with low to medium abundance levels, along with a substantial reduction of the total cost of the experiment. In general, pooling RNA samples or pooling RNA samples in conjunction with moderate reduction of the sequencing depth can be good options to optimize the cost and maintain the power.

## Background

Massively parallel sequencing of cDNA libraries (RNA-seq), is the gold standard for comprehensive profiling of RNA expression [1]. This type of data is used to answer various biological and medical questions, including discovering deferentially expressed (DE) genes between experimental or biological conditions. The use of different biological samples (also known as biological replicates) allow for the estimation of within-group biological variability, which is necessary for making inferences about the conditions under study to reach conclusions that can be generalized [2, 3]. The number of biological replicates in an RNA-seq experiment is typically small because of financial or technical constraints. As a result, statistical tools for testing differential gene expression (DGE) were designed to make efficient use of that type of data. For example, parameter estimations are based on empirical Bayes procedures to share information across genes so that the methods are applicable to small sample sizes [2, 4, 5]. Nevertheless, it is highly recommended to increase the number of biological replicates, especially when there is high biological variability, such that DGE tools deliver their promised performance [6, 7]. Similarly, the sequencing depth (the total number of reads mapped to the reference genome) is another crucial element in the design of DGE studies [2, 3]. For a given budget, it is critical to decide whether to increase the sequencing depth to have more accurate measurements of gene expression levels (especially for low abundant genes) or to increase the number of biological samples with lower average sequencing depth [3, 8].

Situations like budget constraint, lack of sufficient RNA input or large within-group biological variability are common limiting factors in RNA-seq experiments. Under such circumstances, pooling of RNA samples may provide a solution. Pooling of RNA samples takes place by mixing RNA molecules extracted from independent biological samples from the same population (a specific experimental or biological condition), before library preparation. Consequently, pooling results in a smaller number of replicates, and hence lower cost for the subsequent steps. For microarray studies, the adequacy and experimental validation of pooling has been well studied [9–13]. The majority of these studies demonstrate the potential of pooling to tackle budget and technical constraints as well as stabilizing the variability of gene expression measures. For example, Kendziorski et al. [9] demonstrated that the biggest advantage of pooling occurs when the biological variability is large relative to the technical variability. Peng et al. [11] and Shih et al. [10] have also discussed that a properly designed RNA sample pooling scheme can provide adequate statistical power for testing DGE in microarray experiments, while being cost-effective. However, there are also potential limitations of pooling. In addition to the loss of statistical power caused by a small number of pools, it is no longer possible to account for sample-level confounding factors in pooled experiments [10]. For RNA-seq data, Rajkumar et al. [14] have empirically evaluated pooling strategies and concluded that a pooling strategy has limited utility for DGE analysis. However, there is no comprehensive study that thoroughly assessed the adequacy and limitations of RNA sample pooling in RNA-seq experiments, not from a theoretical perspective, nor based on empirical or simulated data pooling.

**Fig. 1:**
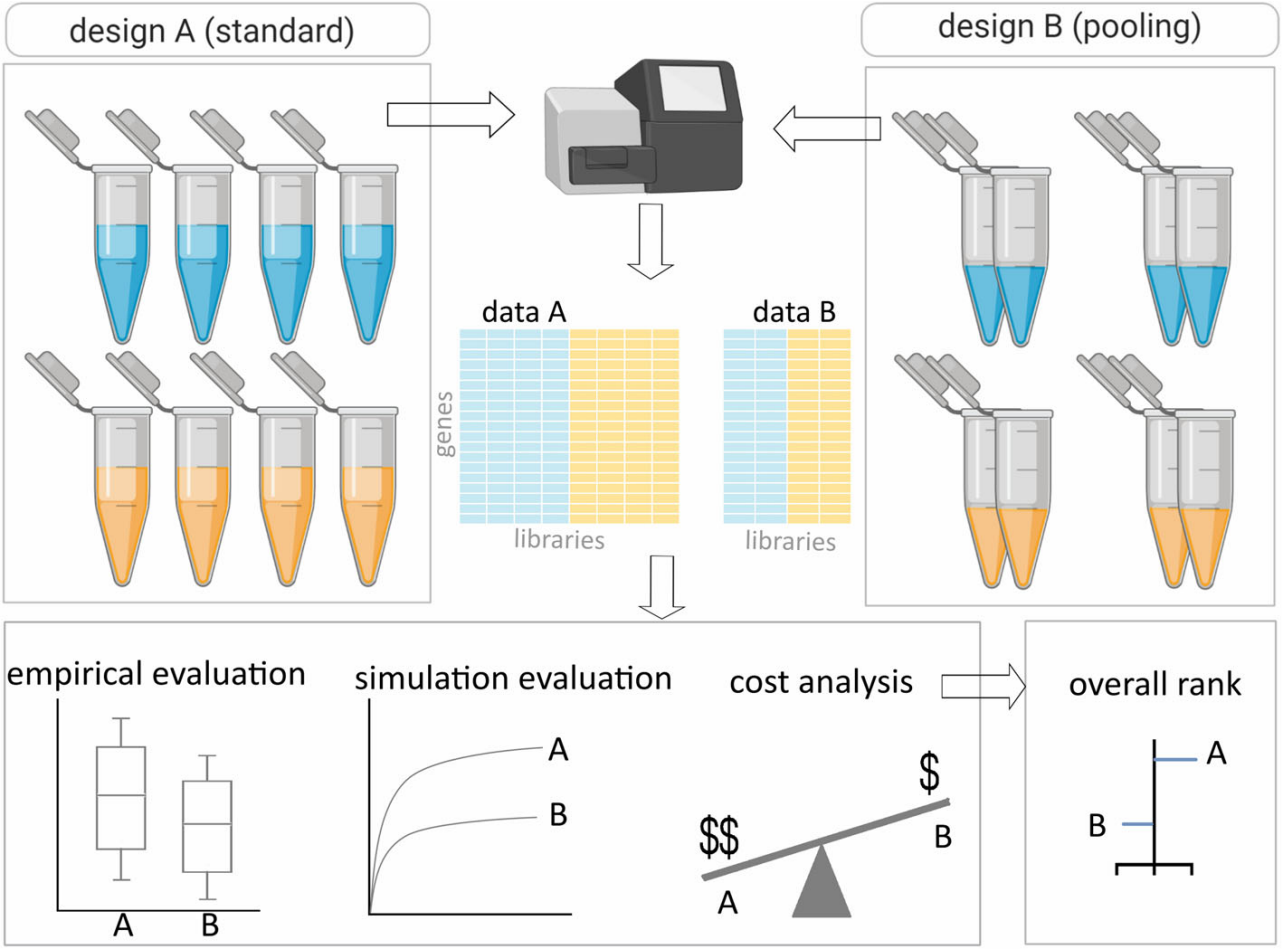
Summary of the workflow. Assessment of RNA sample pooling in RNA-seq experiment involves comparison of standard (design A) and pooled (design B) experimental designs using empirical data, simulated data and total cost assessment. The experimental scenarios are ranked using an overall performance score that summarizes all the comparison metrics

In this study, we evaluate the utility of RNA sample pooling strategies in RNA-seq experiments, using both empirical and simulation methods (Fig. 1). Comparison of systematically chosen varying experimental scenarios enables the evaluation of pooling strategies relative to the standard procedure or reference scenario of unpooled analysis. The empirical assessment is done through analysis of real RNA-seq datasets under various pooling and non-pooling experimental settings. The simulation study is used to rank experimental scenarios with respect to the rate of false and true discoveries in DGE analysis. In addition, we have defined the data generating mechanism in sample pooling strategies from a mathematical perspective for better interpretation of the empirical and simulation results. We conclude that RNA sample pooling can be a cost-effective strategy, provided that the number of pools, pool size and sequencing depth are optimally defined.

## Results

### Data generating model in pooled RNA-seq experiments

A typical RNA-seq experiment consists of three major steps: RNA sample preparation, library preparation, and sequencing. When there is no pooling of RNA samples in the first step (the standard procedure), a library represents a single biological sample. In pooled RNA-seq experiments, a number (*q*) of randomly selected RNA samples are mixed before library preparation and sequencing. As a result, in pooled experiments, a library represents a pool of *q* biological samples. In the subsequent sections, we formalize the RNA sample pooling procedure for better understanding of the data generating process.

Suppose there is no pooling. Let *U*_*gj*_ denote the read count of gene *g*=1,2,…,*G* in biological sample *j*=1,2,…,*n*. To simplify the notations, we focus on a single gene, and hence we drop the subscript *g*. Let the mean and variance of *U*_*j*_ are denoted by *μ*_*j*_=E{*U*_*j*_} and $\sigma ^{2}_{j}=\text {Var}\left \{U_{j}\right \}$, respectively. The objective is to group *n* biological samples from a particular population (condition) into *m* non-overlapping pools (*m*<*n*), each containing *q*>1 unique biological samples. First, we assume the pool size *q* is the same for all pools, and then later we relax this assumption and generalize the theory for pooled experiments with varying pool sizes. To formalize the pooling procedure, we introduce a dummy variable *A*_*jk*_, which is defined as 1 if biological sample *j* is in pool *k*=1,…,*m*, and 0 otherwise. $\mathbf {A}_{j}^{t}=\left (A_{j1},\ldots,A_{jm}\right)$ is the *m*-dimensional vector of indicators for biological sample *j*. We assume **A**_*j*_∼Multinomial(1,(1/*m*,…,1/*m*)). Thus, each biological sample *j* can only be assigned to one pool $\left (\sum _{k=1}^{m} A_{jk}=1 \right)$, and the assignment has probability 1/*m* for all pools. Similarly, we also impose the constraint $\sum _{j=1}^{n} A_{jk}=q$ so that each pool contains exactly *q* biological samples. We further assume that the *A*_*jk*_ are independent of the *U*_*j*_. This assumption makes sense if one randomly assigns the *n* biological samples to *m* pools.

If one aims at a sequencing depth of *L* per pooled library (determined in advance), then pooling of, for example, *q*=2 biological samples A and B with depths *L*_*A*_ and *L*_*B*_, takes place by mixing *w*_*A*_*L*_*A*_ and *w*_*B*_*L*_*B*_ amount of RNA molecules (0≤*w*_*A*_≤1 and *w*_*B*_=1−*w*_*A*_) from sample A and B, respectively. That is, we mix *w*_*A*_ and *w*_*B*_ fractions of the RNA molecules from biological sample A and B, respectively. We consider the mixing weights as random variables and account for their contribution to the variability of the pooled outcome. To formalize this, let the random variable *W*_*jk*_ denote the mixing weight for biological sample *j* in pool *k*. For a given pool *k*, we have a *q*-dimensional vector of these fractions, $\mathbf {W}_{k}^{t} = \left (W_{k1}, W_{k2}, \ldots, W_{kq}\right)$ such that $\sum _{j} W_{jk} =1$. Therefore, if one mixes a proportional amount of RNA samples from each biological sample, then it is reasonable to assume a *q*-component symmetric Dirichlet distribution for mixing weights, i.e. **W**_*k*_∼Dirichlet(**J**), where **J** is a *q*-dimensional vector of 1s. Consequently, the expected proportion of RNA molecules to be pooled becomes E{*W*_*jk*_}=1/*q*. For the previous example of pooling two biological samples A and B, the expected mixing weight is 50% from each sample.

In pooled experiments, *U*_*j*_ are unobservable random variables, and hence we sometime refer to them as virtual counts. Therefore, the data generating model for the observable gene expression measurement *Y*_*k*_ from pool *k*=1,…,*m* with pool size *q*>1 can be written as

1 $$\begin{array}{*{20}l} Y_{k} =\sum\limits_{j=1}^{n} A_{jk} W_{jk} U_{j} + \epsilon_{k},  \end{array} $$

where *ε*_*k*_ is an error term which represents the extra technical variability introduced by the pooling of RNA samples. We assume that *ε*_*k*_ is independent of *A*_*jk*_, *W*_*jk*_ and *U*_*j*_, with *ε*_*k*_∼Normal(0,*σ*^2^).

Model (1) indicates that *Y*_*k*_ is the weighted sum of the virtual counts *U*_*j*_ from the *q* biological samples in pool *k*. Under the assumption that *U*_*j*_, *A*_*jk*_ and *W*_*jk*_ are independent random variables, the expectation of the gene expression measures in pooled sample *k* becomes

2 $$ \begin{aligned} \mathrm{E}\left\{{Y_{k}|J_{k}}\right\} = \frac{1}{q}\sum\limits_{j\in J_{k}}^{q} \mu_{j}, \end{aligned}   $$

where *J*_*k*_ is the set the indices *j* for biological samples included in pool *k* (i.e. *J*_*k*_={*j*: *A*_*jk*_=1}). This indicates that the expected gene expression measurements in a particular pool is equal to the average of the expected expression levels from the *q* biological samples included in that pool. The variability of the gene expression levels in pool *k*, accounting for the sampling variability (**A**_*j*_), becomes

3 $$ \begin{aligned} \text{Var}{Y_{k}} = \frac{2}{n(q+1)}\sum\limits_{j=1}^{n} (\mu^{2}_{j}+\sigma^{2}_{j}) -\frac{1}{n^{2}}\sum\limits_{j=1}^{n}\mu_{j}^{2} + \sigma^{2}. \end{aligned}   $$

**Fig. 2:**
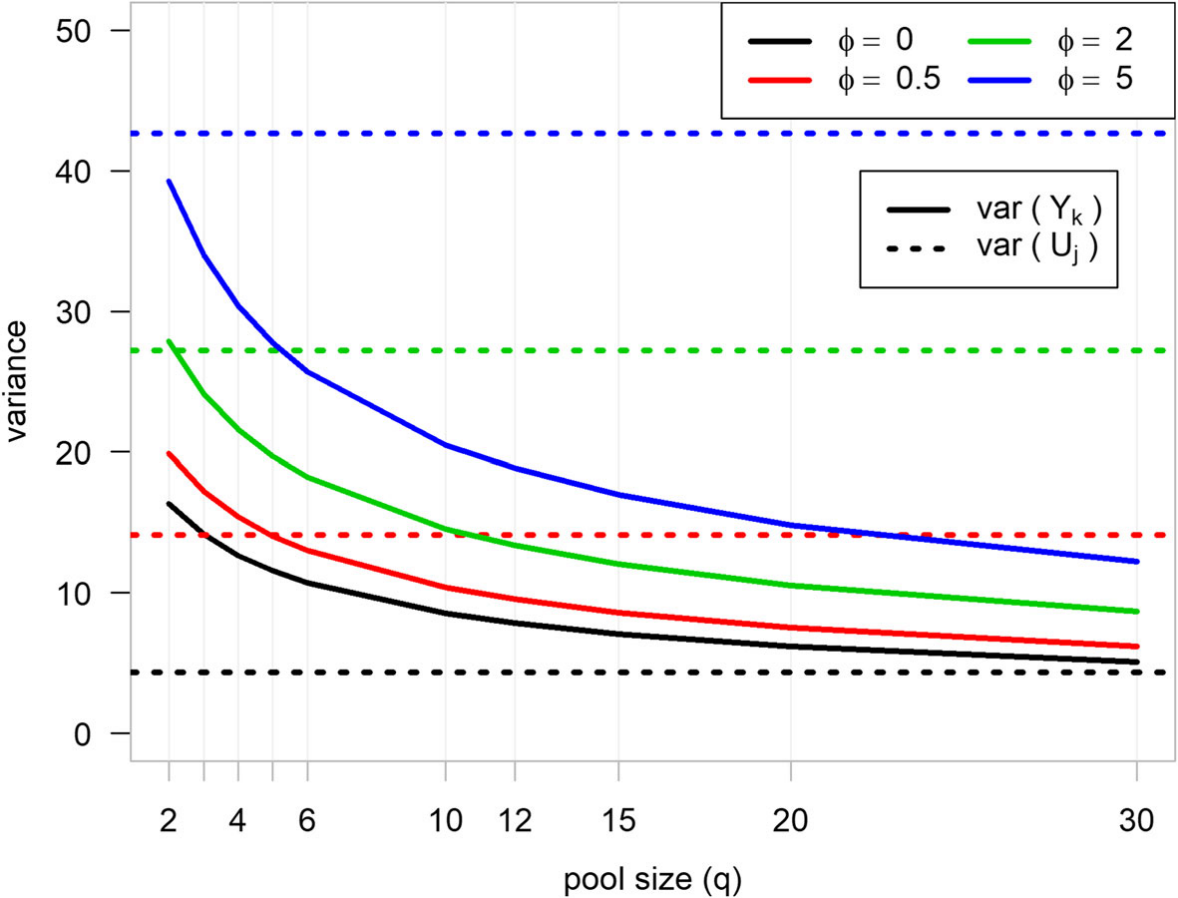
Variance at different pool sizes. The variance of the gene expression levels from pooled and non-pooled experiments. In particular, the virtual counts *U*_*j*_ were generated from a negative binomial distribution with mean *μ*_*j*_ and over-dispersion parameter *ϕ*. $\mu _{j} = \rho L_{j}^{0}$, where *ρ* is the relative abundance (*ρ*=10^−6^), and $L_{j}^{0}$ is the virtual library size in biological sample *j*, and $L_{j}^{0}$ are uniformly sampled between 15−25×10^6^. *Y*_*k*_ is the outcome from a pooled design with a pool of size *q* according to the model in (1)

The proof is available in Section 1.1 of the Supplementary file, with empirical confirmation by Monte-Carlo simulations (see Supplementary Fig. S1). Eq. 3 indicates that Var{*Y*_*k*_} is inversely proportional to the pool size *q*, suggesting that pooling reduces the variability of the gene expression measurements, given *σ*^2^ is sufficiently small. However, the amount of variability reduction depends on the level of variability among the *U*_*j*_ (Fig. 2). In particular, for large $\sigma _{j}^{2}$, a small pool size, such as *q*=2, is sufficient to reduce the variability. Note that this variability is the within-group variability as pooling independently takes place within each group.

The mean expression of a gene $\bar {Y}$ from a pooled experiment is an unbiased estimator of the true mean expression similar to that of the standard experiment $\left (\text {i.e.}\, \mathrm {E}\left \{{\bar {Y}}\right \}= \mathrm {E}\left \{{\sum _{k=1}^{m}Y_{k}/m}\right \}= \mathrm {E}\left \{{\bar {U}}\right \} =\frac {1}{n}\sum _{j=1}^{n} \mu _{j}\right)$. Furthermore, we examine the effect of pooling on the estimation of the relative abundance *ρ* of a gene and the log-fold-change (LFC) between two independent groups. The LFC is a quantity that is commonly used to calibrate the biological effect of interest. The LFC is defined as $\theta =\log _{2}\frac {\rho _{2}}{\rho _{1}}$, where *ρ*_1_ and *ρ*_2_ are the relative abundances in groups 1 and 2, respectively. Although pooling results in expression levels with a lower variance, the variance of the estimates of the relative abundance ($\hat \rho $) and the LFC between two independent groups $\left (\hat \theta \right)$, have a variance that is at least 2*q*/(*q*+1) times higher than that of the estimates from standard experiments (see Section 1.2 of the Supplementary file for details). This is the direct consequence of the reduction of the number of replicates in pooled experiments. Consequently, the statistical power of testing the null hypothesis *H*_0_:*θ*=0 (no DGE) against the alternative *H*_*A*_:*θ*≠0 at *α* level of significance can be lower in pooled experiments than in standard experiments (the full budget experiment). Based on the negative binomial assumption for the virtual counts *U*_*j*_, we can determine the statistical power of testing the above hypothesis for a particular gene [15]. That is, given the number of RNA samples in groups 1 and 2 (*n*_1_ and *n*_2_, respectively), pool size *q*, the LFC to be detected *θ*, and the over-dispersion *ϕ*, the power of the two-sided likelihood-ratio test at significance level *α* can be calculated as,

4 $$ \text{power} \;\le \; \Phi\left\{\frac{\sqrt{n_{1}(q+1)}|\theta|-Z_{\alpha/2}\sqrt{2qV_{0}}}{\sqrt{2qV_{A}}}\right\},   $$

where *Φ*(.) is the standard normal cumulative distribution function, *Z*_*α*/2_ is the (1−*α*/2)100*%* quantile of the standard normal distribution, and *V*_0_ and *V*_*A*_ are the variances of the LFC estimate $\left (\hat \theta \right)$ under *H*_0_ and *H*_*A*_, respectively. The details of the power calculation can be found in Section 1.3 of the Supplementary file.

**Fig. 3:**
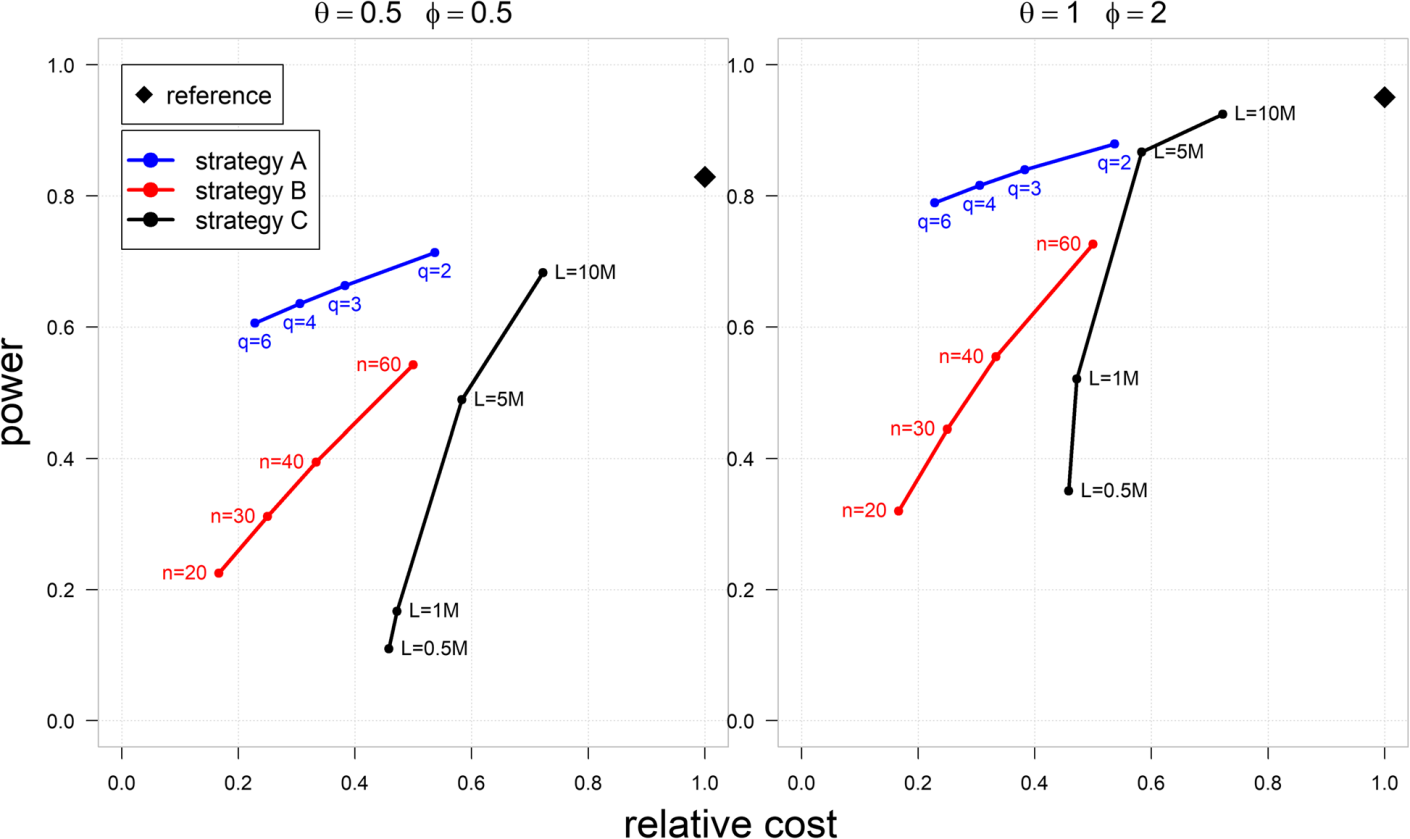
Zodiac plot representing the trade-off between power and cost. The zodiac plot shows the statistical power (at 5% significance level) to call a single gene DE versus the relative total cost of data generation for three different cost-saving strategies compared to a reference design. The power is calculated for a gene with relative abundance *ρ*=10^−7^ in one group, LFC (‘effect size’) *θ*∈{0.5,1}, and over-dispersion (‘variability’) *ϕ*∈{0.5,2}. The reference design consists of 120 samples (*n*_1_=*n*_2_=60) with average library size of 20M per sample and no pooling. Strategy A is pooling with pool size *q*∈{2,3,4,6} and average library size of 20M per pool. Strategy B is similar to the reference, except the number of samples is reduced to *n*∈{60,40,30,20}. Strategy C is similar to the reference, except the sequencing depth is reduced to *L*∈{10*M*,5*M*,1*M*,0.5*M*}. The relative cost is calculated as the total cost of a particular strategy divided by that of the reference design

In Fig. 3 and Supplementary Figs. S2–S4, we presented the relationship between the power and the total cost of the data generation for different experimental designs, including RNA sample pooling. In particular, we compare three cost-saving strategies (sample pooling, shallow sequencing depth, and reducing sample size) with respect to the power and the relative cost compared to a reference scenario (full budget experiment). Further details are in Section 1.3 of the Supplementary file. Moderate reduction of the sequencing depth without reducing the number of replicates seems better in maintaining the power (the power that would be achieved using the reference design) with lower sequencing cost. However, this strategy is less effective for low-abundance genes (Fig. 3, Supplementary Fig. S2). This result is in line with a previous study [8] that demonstrated that the number of replicates is more important than the sequencing depth to maintain the power, particularly for moderate to highly expressed genes. It is also essential to note that the power calculation (4) does not take into account the library size variability, which may compromise the power of the test [6]. Of note, pooling seems to be an effective strategy to maintain the power and reduce the cost, especially for low and moderately expressed genes (Fig. 3, Supplementary Figs. S2 and S3). For pooling strategies, a small pool size is more effective in preserving the power when there is large variability (high over-dispersion). The third strategy, reducing the number of replicates, is generally worse in terms of power, yet it reduces the total cost significantly. In summary, an RNA sample pooling strategy can be a good choice to optimize the power and data generation cost, especially when many of the genes are expressed at low or medium levels like long-non-coding RNAs [6] with a substantial reduction of the library and sequencing costs. Of note, for gene expression levels with a small biological variability (represented by a negative binomial dispersion *ϕ*=0.5) and large LFCs (*θ*=1), all strategies seem to be equally effective. In such scenarios, it can be suggested that reducing the number of samples (strategy B) or pooling with a large pool size can be used to optimize the cost with comparable power to the reference design.

The same conclusion can be drawn when different pool sizes are used across pools. That is, let *q*_*k*_ denote the pool size in pool *k*, then the variance of the LFC estimate in the pooled experiment $\hat \theta ^{*}$ becomes at least $\frac {2n}{m^{2}}\sum _{k=1}^{m}\left (\frac {1}{1+q_{k}}\right)$ times higher than that of the estimates from standard experiment. As a result, the same power function (4) can be used with the constant *q* is substituted by the fraction *n*/*m*, where, as defined earlier, *m* and *n* are the number of pools and RNA samples in a given group, respectively.

### Experimental scenarios

To evaluate the pooling strategy compared to the standard procedure, two sets of scenarios were investigated, one starting from the tumor tissue RNA-seq data and one from the cell line RNA-seq data, representing typical data with high and low within-group variability, respectively [6].

The first set comprises a total of 12 test scenarios and one reference scenario (Table 1a). The reference scenario represents a standard tissue RNA-seq experiment without pooling consuming a maximum budget in terms of the number of samples, number of libraries, and sequencing depth. The 12 test scenarios include a unique combination of the number of RNA samples, sequencing depth, number of libraries, and pool size (q). Consequently, the data generation cost (total cost of RNA sample preparation, library preparation and sequencing) is different for each scenario. In particular, the reference scenario contains a subset of 80 high-risk neuroblastoma samples forming two groups: the MYCN amplified (*n*_1_ = 40) and MYCN non-amplified (*n*_2_ = 40). The average sequencing depth per sample in this data is approximately 20 million reads with a range 11−30×10^6^. Subsequently, the data for the test scenarios were generated from the reference scenario according to the data generation model in (1).

**Table 1:** Summary of RNA-seq experimental scenarios

Scenario	Number of	Number of	Total reads	Total cost	≈ depth per	Number of	Pool size	RNA
	RNA	libraries	counts		library ×10^6^	libraries		pooling
	samples		×10^6^		(min – max)	per group		
**a**Scenarios based on the Zhang neuroblastoma samples								
A0 (reference)	80	80	1600	€ 21,800.00	20 (11.2–30.0)	40	-	No
A1	40	40	800	€ 10,800.00	20 (11.2–29.7)	20	-	No
A2	40	40	400	€ 7,800.00	10 (4.9–13.1)	20	-	No
A3	80	80	800	€ 15,600.00	10 (5.0–13.5)	40	-	No
A4	80	80	400	€ 12,600.00	5 (2.5–6.7)	40	-	No
B1	80	40	800	€ 11,600.00	20 (11.8–28.3)	20	2	Yes
B2	40	20	400	€ 5,800.00	20 (13.4–28.7)	10	2	Yes
B3	80	40	400	€ 8,600.00	10 (5.3–12.7)	20	2	Yes
B4	40	20	200	€ 4,300.00	10 (6.0–12.9)	10	2	Yes
C1	80	20	400	€ 6,600.00	20 (15.00–27.8)	10	4	Yes
C2	40	10	200	€ 3,300.00	20 (14.7–26.0)	5	4	Yes
C3	80	20	200	€ 5,100.00	10 (6.7–12.5)	10	4	Yes
C4	40	10	100	€ 2,550.00	10 (6.6–11.7)	5	4	Yes
**b** Scenarios based on the NGP neuroblastoma cell lines								
A0 (reference)	18	18	270	€ 4,185.00	15 (14.3–19.3)	9	-	No
A	6	6	90	€ 1,395.00	15 (15.0–17.7)	3	-	No
B	12	6	90	€ 1,515.00	15 (14.9–17.6)	3	2	Yes
C	18	6	90	€ 1,635.00	15 (15.3–17.9)	3	3	Yes

The total data generation cost of a particular scenario is given by (*S*×20)+(*L*×100)+(*R*×7.5), where S is the number of RNA samples (with RNA preparation cost €20.00 per sample), L is the number of libraries (with library preparation cost €100.00 per library), R is the total sequencing depth (with cost €7.50 per 1 million sequencing reads)

The second set of experimental scenarios constitutes of three test scenarios generated with the cell line RNA-seq data (Table 1b). These scenarios enable us to explore the utility of pooling strategies in experiments in which the biological variability is typically low. The three scenarios consist of 3 sequencing libraries per treatment group, derived from either single (unpooled) or pooled RNA samples (2 or 3 per library). A reference scenario with 9 RNA samples per treatment group without pooling is also included.

The experimental scenarios in Table 1a represent different cost-saving strategies in RNA-seq experiments. In particular, reducing the number of RNA samples (scenario A1), reducing both the number of RNA samples and sequencing depth (scenario A2), reducing the sequencing depth (scenarios A3 and A4), pooling of RNA samples (scenarios B1 and C1), pooling and reducing the number of RNA samples (scenarios B2 and C2), pooling and reducing the sequencing depth (scenarios B3 and C3), and both (i.e. pooling, reducing the sequencing depth and reducing the number of RNA samples, scenarios B4 and C4). Similarly, the scenarios in Table 1b represent cost-saving strategies by pooling of RNA samples with different pool sizes.

### Empirical evaluation of pooling RNA samples

Using the Zhang and NGP nutlin RNA-seq datasets, we empirically compared the experimental scenarios in Table 1 (a and b). In particular, we focus on comparing the distribution of the mean and variability of normalized gene expression levels, the LFC estimates, and the number and characteristics of genes called DE at 5% nominal FDR level.

The varying sequencing depth across scenarios resulted in different numbers of genes with sufficient expression level (i.e. the non-zero counts in at least 3 samples, Supplementary Fig. S5). From a cost perspective, the pooling scenarios generally have lower cost with relatively higher number of sufficiently expressed genes, compared to that of non-pooling scenarios (Table 1 and Supplementary Fig. S5). Besides, the sample level exploratory data analysis shows that the degree of similarity between samples (in terms of correlation) increases with increasing pool size (Supplementary Fig. S6). The two-dimensional visualization of the neuroblastoma samples (for each scenario) using principal component analysis also shows that the within-group variability is smaller than the between-group variability in pooled experiments, where group is here the MYCN status (Supplementary Fig. S7). On the other hand, pooling may not help to reduce the frequency of zero counts per sample, as this characteristic is mostly related to the sequencing depth (Supplementary Fig. S6).

**Fig. 4:**
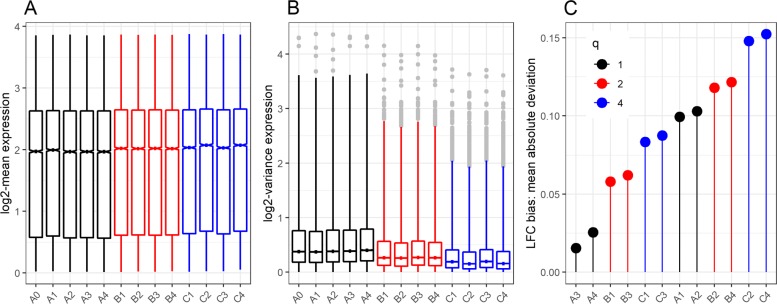
Empirical results. **a**–distributions of the average normalized counts per genes (in log2 scale), **b**–distributions of the variability of normalized counts per gene (in log2 scale), and **c**–The LFC bias in terms of the mean absolute difference with the LFC estimate from the reference scenario (A0)

The distribution of gene-specific average expression is the same for all scenarios (Fig. 4-panel A). This result is in line with the theoretical result that pooling results in an unbiased estimate of the average gene expression level even for different choices of pool size. In contrast, the observed variance was lower for pooling scenarios (Fig. 4-panel B). This result also supports the theoretical results in (3) that the variability decreases with increasing pool size *q*.

We also evaluated the bias of the LFC estimates in each test scenario relative to the estimates from the reference scenario. In particular, the mean absolute difference (MAD) for scenario *s* is calculated as MAD$_{s} = G^{-1}\sum _{g=1}^{G}|LFC_{gs} - LFC_{g0}|$, where *LFC*_*gs*_ and *LFC*_*g*0_ are the LFC estimate for gene *g* from test scenario *s* and the reference scenario (A0), respectively. MAD _*s*_ evaluates the risk associated with using scenario *s* in terms of losing DE signals that would be detected at the full budget design (A0). The observed LFC bias was generally low across the scenarios (Fig. 4-panel C). A small number of replicates, however, tends to result in a higher LFC estimate bias compared to a lower sequencing depth per library. The magnitude of bias caused by the reduction in the number of replicates is relatively smaller for pooling scenarios than for non-pooling scenarios. For example, pooling scenarios C1 and C3 (that have 10 libraries per group and 20M and 10M depth, respectively) resulted in lower bias than that of the non-pooling scenario A1 and A2 (that have 20 libraries per group with 20M and 10M depth, respectively). On the other hand, pooling scenarios B2, B4, C2 and C4 resulted in the largest bias, which can be explained by the highest reduction of the number of replicates.

Reducing the within-group variability by pooling RNA samples may enhance the resolution of the biological effect. This can be seen from the standardized LFC estimate $\left (LFC/\hat \sigma (LFC)\right)$, which is also known as the signal-to-noise ratio. The absolute standardized LFC increases with increasing sample size and decreasing variance. We compared the scenarios with respect to the standardized LFC estimates for a subset of genes. Two particular subsets of genes were considered: MYCN pathway genes – known to be DE between the 2 groups [16], and the top 200 DE genes detected in the reference scenario. The result (Supplementary Fig. S8) shows that the standardized LFC estimates from scenarios A3 and A4 are the lowest followed by pooling scenarios B1, B3, C1 and C3 (for MYCN pathway) and B1 and B3 (for the top 200 DE genes). Scenario A3 and A4 contain the maximum numbers of libraries, and hence they resulted in estimates close to the reference scenario. On the other hand, the pooling scenarios B1, B3, C1 and C3 have lower variability and hence resulted in estimates almost close to that of the reference scenario, but with fewer libraries.

From the DGE analysis, both limma-voom and edgeR called the largest number of genes DE (at 5% nominal FDR) in the reference scenario, followed by pooling scenarios B1, B3, C1, and C3 (limma-voom) and B1, A3 and A1 (edgeR) (Supplementary Fig. S9-panel A). To gain a rough insight in the number of false and true positives, we use the level of *concordance* with the reference scenario (defined as the fraction of genes called DE in test scenario that are also called DE in the reference scenario). Scenarios A3 and A4 from edgeR, and scenario A3, A4, B1, B3, C1 and C3 from limma-voom, resulted in more than 75% concordance (Supplementary Fig. S9-panel B). Overall edgeR tends to call a large number of genes DE with lower concordance level compared to that of limma-voom. For limma-voom, more than 87.5% of concordance was achieved with scenarios A3, A4 and B1, whereas less than 50% concordance was observed from pooling scenarios B4, C2 and C4. These results indicate that with moderate depth per sample and number of replicates per group (e.g. scenario B3 (*q*=2) with 10M reads per library and 2 ×20 replicates or C1 (*q*=4) with 20M reads per library and 2 ×10 replicates) one can increase the chance of recovering the DE genes that would be detected with the full budget design. We will formally examine the false and true positive proportions in a simulation study in the subsequent section. Furthermore, the characteristics of the genes that are exclusively called DE in each test scenario are quite different. Pooling scenarios tend to favor low-abundance genes with high coefficients of variation (as determined based on the data from the reference scenario). In contrast, the non-pooling scenarios are biased towards highly expressed genes with small variability (Supplementary Fig. S10). This result is in agreement with the theoretical argument discussed above that pooling strategies are generally robust for low and medium expressed genes compared to designs with shallow sequences (such as scenario A3 and A4).

The results from the comparison of the second set of experimental scenarios with the NGP nutlin dataset (see Table 1b) show that pooling did not have much effect on the overall result. In particular, unlike the pooling scenarios based on the Zhang data, the variability of the gene expression data did not change across the scenarios (Supplementary Fig. S11-panel A). The gene expression levels in the NGP nutlin dataset present with low variability. Consequently, in line with the theoretical results, a large pool size is required to reduce the variability of the virtual counts. Only a small reduction of the LFC estimation bias was observed for the pooling scenarios than for the non-pooling scenario (Supplementary Fig. S11-panel B). The number of detected DE genes (at 5% nominal FDR) and the concordance level were nearly the same across scenarios (Supplementary Fig. S12). However, it is worth noting that the difference in the concordance level between edgeR and limma-voom reached up to 40%. Approximately 65% of concordance was observed for edgeR, whereas limma-voom achieved approximately 99% concordance (Supplementary Fig. S12-panel B). From a cost perspective, a pooling strategy seems not effective in this case. In addition, we compared the power-cost trade-off for designs representing the scenarios in Table 1b under the negative binomial assumption. In particular, the power-cost trade-off was assessed for designs with equal number of replicates (3 replicates per group), that is, 3 individual cell line samples (scenario A), 3 pools of 2 cell line samples (*q*=2, scenario B) and 3 pools of 3 cell line samples (*q*=3, scenario C). The results indicate that scenarios B and C come with higher power but also with somewhat higher cost (due to extra RNA preparation) compared to scenario A. The benefit is especially true for low abundant genes with a small LFC (*θ*) (Supplementary Fig. S13). For medium or high abundant genes with higher LFC (*θ*≥1), cell line sample pooling is not cost-effective.

### Simulation based evaluation of pooling RNA samples

Next, we run a simulation study to determine the actual false discovery rate (FDR) and true positive rate (TPR, or sometime called sensitivity) associated with testing of DGE in the experimental scenarios shown in Table 1a. RNA-seq datasets were simulated with built-in truths using the *SPsimSeq* R package (v1.0.0) [17]. *SPsimSeq* simulates realistic RNA-seq data from a semi-parametrically constructed distribution of gene expression levels from a real RNA-seq data (see the Methods section for the implementation of the simulation).

**Fig. 5:**
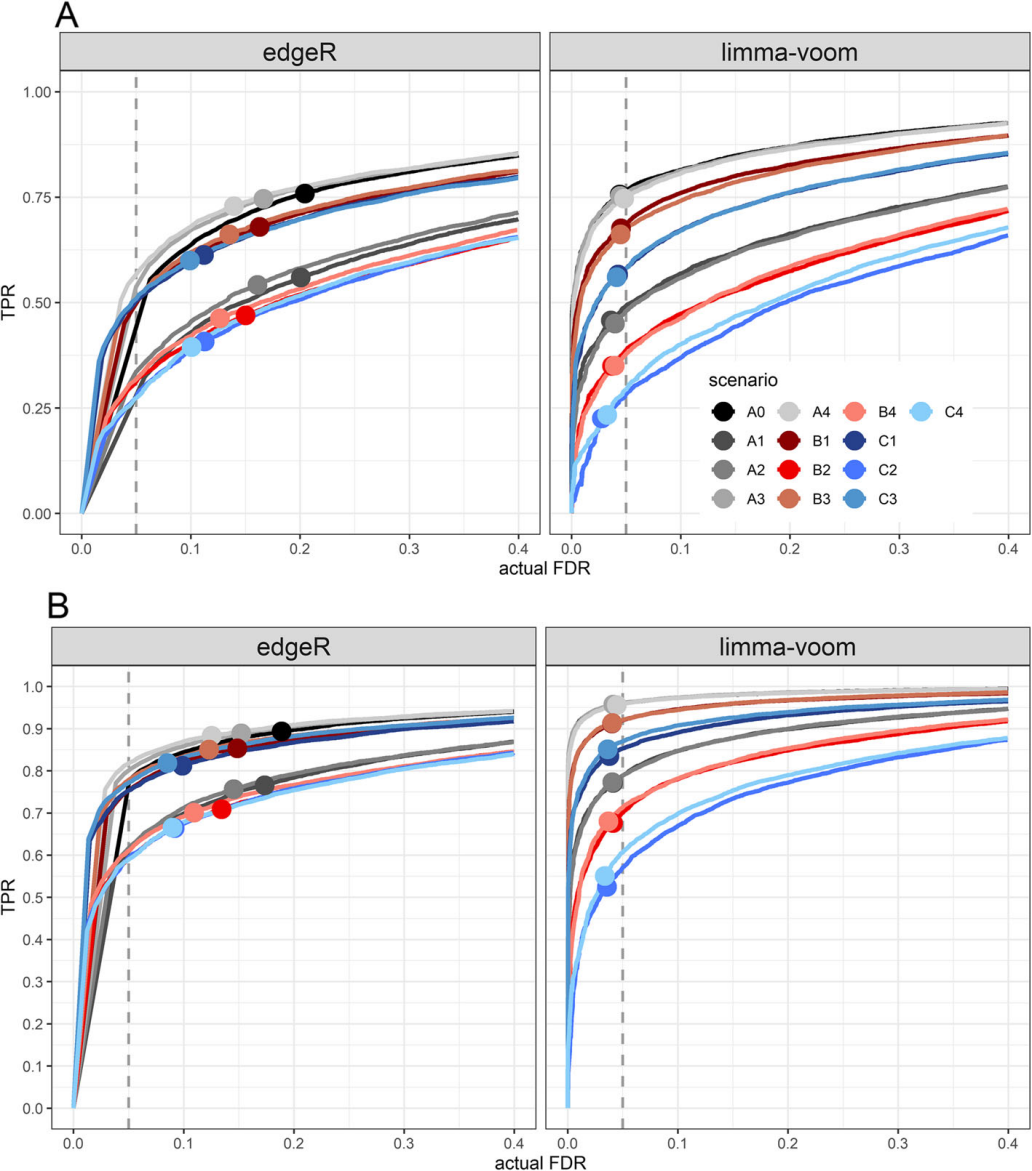
Simulation results. Results of the simulation based evaluation: The curves show the trade-off between the true positive rate (TPR) and the actual FDR evaluated at 0-40% nominal FDR level. The solid circles on each curve indicate the TPR and actual FDR at 5% nominal FDR (indicated by the vertical dashed line). The DE genes in the simulated dataset have |*LFC*|≥0.5**a** or |*LFC*|≥1**b**

The simulation results (Fig. 5) indicate that the scenarios markedly differ with respect to the sensitivity (TPR) of detecting true DE genes, for both edgeR and limma-voom. The actual FDR rate for limma-voom is equally well controlled across all scenarios, whereas edgeR showed variable and overall weak FDR control. The maximum sensitivity was attained with the reference scenario and scenario A3 and A4 for both DGE tools and across all simulation settings.

The level of sensitivity for limma-voom differs among scenarios quite substantially with a range 20-75% and 55-95% at the 5% nominal FDR when the LFC of the DE genes is greater than 0.5 and 1, respectively. In particular, scenarios with equal number of libraries and pool size resulted in almost the same sensitivity, regardless of the sequencing depth difference. For example, scenarios B1 and B3 or C1 and C3 or A1 and A2, attained an equivalent level of sensitivity. For pooling scenarios B1, B3, C1 and C3, higher sensitivity is observed than for the non-pooling scenarios A1 and A2, even though A1 and A3 have twice the number of libraries than C1 and C3, or equal number of libraries to B1 and B3. This result points at the utility of an RNA sample pooling strategy to balance the number of replicates and variability. For edgeR, scenarios A0, A3, A4, B1, B3, C1 and C3 show comparable sensitivities for both LFC thresholds. Although the actual FDR is overall not well controlled by edgeR, it is relatively lower for the pooling scenarios B1, B3, C1, and C3 with maximal sensitivity.

Furthermore, for limma-voom, among the pooling scenarios, B1 and B3 are less than 10% off from the maximum sensitivity achieved with the reference scenario (Fig. 5-panel A). This offset further reduces to less than 5% when the LFC threshold is at least 1 (Fig. 5-panel B). Similarly, pooling scenarios C1 and C3 resulted in less than 20% and 10% lower sensitivity than that of scenario A0 for a LFC threshold of 0.5 and 1, respectively. In general, the magnitude of the LFC for the simulated DE genes showed considerable effect on the sensitivity (for both edgeR and limma), such that for simulations with high LFC for DE genes, the performance-gap between scenarios narrows down. This result suggests that pooling strategies in RNA-seq experiments have the potential to replace the full design experiment if one looks for DE genes with a high magnitude of biological effects.

Generally, the simulation and empirical results align with each other and support the theoretical predictions in the sense that the reduction in the number of replicates (libraries) comes with the cost of losing sensitivity. However, both results demonstrate that pooling strategies compensate the performance offset caused by sample size reduction. In addition, the choice of DGE tool is a critical factor for DGE analysis with pooled experiments. For example, edgeR focuses on maximizing the sensitivity to detect true DE genes with liberal performance in terms of the FDR control. Therefore, if one aims at only highlighting all possible candidate DE genes, then edgeR can be a good choice for pooling experiments with relatively flexible choice of pooling designs. On the other hand, limma-voom guarantees control of the FDR for all design choices but its sensitivity is strongly dependent to the number of replicates. Therefore, if one aims at maximizing the sensitivity with the actual FDR within the tolerance range and pooling does not result in too much reduction of the sample size, then limma-voom is a good choice for pooled experiments.

### General summary of the empirical and simulation results

**Fig. 6:**
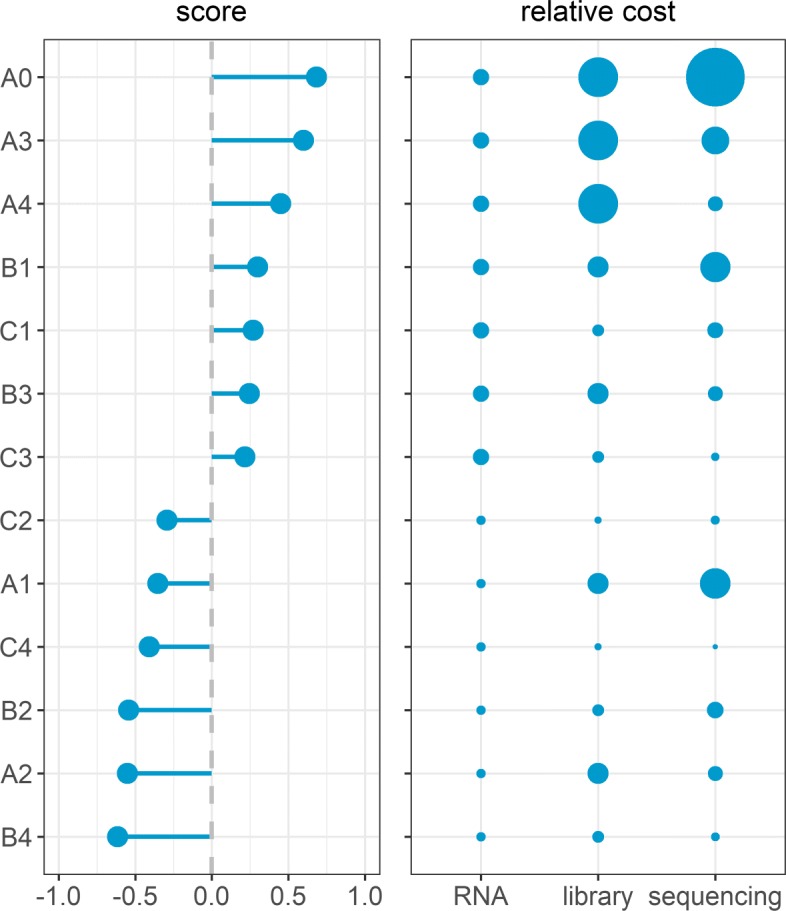
Ranking of experimental scenarios based on the overall performance and cost. Performance ranking of RNA seq experiment design scenarios. Ranks are determined using a score that summarizes the overall performance of scenarios using empirical and simulated RNA seq data. In particular, five metrics were summarized: the inverse of the LFC estimate bias, standardized LFC for MYCN geneset (absolute value), concordance with reference scenario, one minus the actual FDR (at 5% nominal FDR level), and sensitivity (at 5% nominal FDR level). These metrics are standardized across scenarios, and then scenarios are ranked based on the average standard score across the metrics. The solid circles indicate the relative data generation cost of RNA sample preparation, library preparation and sequencing (relative to the corresponding cost from the reference scenario)

The experimental scenarios in Table 1a are ranked based on a score that summarizes the empirical and simulation results (see Fig. 6). In particular, five metrics were summarized: the inverse of the LFC estimate bias, standardized LFC for MYCN geneset (absolute value), concordance with reference scenario, one minus the average actual FDR (at 5% nominal FDR level), and sensitivity (at 5% nominal FDR level). These metrics are standardized across scenarios, and then scenarios are ranked based on the average standard score across the metrics. Higher ranks indicate better performance. Among the non-pooling scenarios, A3 and A4 outperformed all other test scenarios. The theoretical results have demonstrated that the strategy of reducing only the sequencing depth (equivalent to scenarios A3 and A4) is effective in recovering the power and reduce sequencing cost mostly for medium to highly expressed genes, which is the case for the Zhang data. However, from a cost perspective, these scenarios reduce only the sequencing cost (Fig. 6). Also, as the theoretical and empirical results show, these strategies (A3 and A4) are not generally effective for genes with low and medium levels of expression. This can be seen by the fact that because of the library size reduction, scenarios A3 and A4 resulted in a substantially smaller number of genes with sufficient level of expressions compared to that of all the remaining scenarios (Supplementary Fig. S5, see for example C1 and C3 vs. A3 and A4). The pooling scenarios B1, C1, B3 and C3 are ranked above the average, with lower library preparation and sequencing costs and with higher number of sufficiently expressed genes compared to that of A3 and A4. In contrast, pooling scenarios C2, C4, B2 and B4 and the non-pooling scenarios A1 and A2, showed worst overall performance.

In general, the difference in the number of libraries appeared to be a critical factor that leads to the overall performance difference between scenarios (Supplementary Fig. S14-panel A). For example, scenarios A2 and A3 have equal sequencing depth per library (approximately 20×10^6^) but different numbers of libraries (40 and 80, respectively). As a result, A2 is seven ranks below A3 (Fig. 6). Similarly, a wide rank gap is observed between scenarios A0 and A1, B3 and B4, B1 and B2, C3 and C4, and C1 and C2. It is worth noting that the performance gap because of sample size difference is smaller for pooled scenarios than for non-pooled scenarios. In addition, given an equal number of libraries and sequencing depth per library, pooling scenarios (larger pool size) improve the overall performance (Supplementary Fig. S14-panel C). For example, B1 is five ranks ahead of A1, and B3 is six ranks ahead of A2. It can also be seen that C1 is better than B2 and B3, and C3 is better than B2 and B4; implying that increasing pool size improves the overall performance. In contrast, the difference in sequencing depth per library showed a slim effect on the overall score (Supplementary Fig. S14-panel B).

## Discussion

The strategy of pooling RNA samples in gene expression studies, especially in microarray studies, has been shown to have the potential to optimize both the cost of the data generation process as well as the statistical power for testing DGE [9–13]. Given the very different nature of RNA-seq data and biases, we have explored the utility of pooling RNA samples in RNA-seq experiments using several performance evaluation metrics and experimental scenarios. We started by mathematically describing the data generation model for pooled experiments. The model accounts for the additional sources of variability caused by the pooling strategy, such as the random assignment of biological samples to pools and the random mixing weights of RNA samples. The model indicates that pooled RNA-seq designs result in unbiased gene expression measures with reduced within-group variability. Therefore, pooling has the potential to balance variability and sample size to detect the biological effects of interest. Similar conclusions had been reached by studies that assessed the utility of pooling strategies in microarray experiments [9–11, 13], such that a pooling strategy is particularly useful when there is a large heterogeneity within a population apart from cost-efficiency. However, the model (1) also indicates that estimates of statistics that quantify the biological effect, such as the LFC, may have relatively higher variance resulting from the reduction in the number of replicates by pooling of biological samples. The extra variability of these statistics is less for smaller pool sizes. Consequently, the statistical power of testing for DGE using pooled experiments can be lower than that of the full budget design unless a proper pool size is chosen. Specifically, given there is a sufficient number of RNA samples, a small pool size such as *q*=2 is sufficient to stabilize the large variability among the gene expression levels and optimize the trade-off between the power and the data generation costs. In contrast to the typical cost-saving strategies (lower sequencing depth or a smaller number of biological samples), pooling strategies are robust and cost-effective for genes with low to moderate level of expression, for which most statistical methods fail to perform optimally [6]. The model makes use a few modest assumptions, for instance that the mixing weights *W* in a given pool follow a Dirichlet distribution. Of note, the parameters of the Dirichlet distribution, *α*_1_,…,*α*_*q*_, are all set to 1, resulting in maximum variability representing the worst-case-scenario. The independence assumption between the allocation of RNA samples to pools (denoted by a random variable *A*) and the gene expression levels *U* is not a strong assumption either, and can be effectively achieved by random allocation of RNA samples to pools. Similarly, the assumption between *W* and *U* holds if the mixing weights in a pool are determined independently of the transcriptome size in each biological sample. As sample pooling should be done at the RNA level, the transcriptome size is not know in advance, and hence, pooling will be random and independent. Consequently, it is reasonable to assume that the random variables *A*, *W* and *U* are independent that drive the mean and variance of a pooled outcome in (2) and (3), respectively.

The adequacy of pooling RNA samples in RNA-seq experiments was subsequently assessed based on both empirical and simulated RNA-seq datasets. For this purpose, a variety of experimental scenarios were compared with a reference design, using several performance evaluation metrics. Besides cost reduction, our findings suggest that pooling strategies offer a number of benefits. In particular, pooling reduces the within-group variability that enables detecting biological effects with a small sample size (the number of pools), hence lower library preparation and sequencing costs. We have also shown that the high level of noise associated with low-abundance genes, which challenge statistical tools for testing DGE, can be mitigated by pooling RNA samples. Pooled experiments can be valid alternatives for DGE analysis, in which the objective is highlighting genes with strong biological effect, such as large LFC. However, pooling in general does not guarantee better results unless the key elements of the pooling experiment are carefully chosen. That is, for pooling to be equally effective to the standard RNA-seq experiment, it is essential to carefully determine the pool size, the number of pools, and sequencing depth depending on the level of variability and the number of RNA samples. The choice of a statistical tool for testing DGE is another essential part of designing pooled experiments. One of the apparent drawbacks of pooling experiments is the reduction of the number of replicates, which most statistical methods strongly rely on for optimal performance. However, our results demonstrate that pooling has the potential to compensate for the loss of the number of replicates by reducing the within-group variability unless the pooling strategy results in too much reduction of the number of replicates. Of note, pooling might not be beneficial when the gene expression levels display low variability, as, for example, in experiments with cultured cells.

One limitation of our study is that the demonstrated utilities of a pooling strategy were based on proper pooling and sub-sampling from a real read count matrix from a particular experimental design. In practice, however, pooling experiments would involve pooling of the RNA molecules before library preparation, and hence extra technical variability resulting from pooling could be anticipated. This extra variability is represented in the data generation model (1) by an additive random error term *ε*_*k*_, which, however, showed a negligible effect on the statistical power for testing DGE. Finally, we wish to note that one single experiment with pooling of RNA samples does not have the capacity to confirm or contradict the theoretical findings in our study. In reality, a few dozen experiments should be performed, but this is beyond the scope of this study. We recommend further research with real pooling of RNA samples to verify the theoretical results presented in our computational study.

## Conclusions

We have shown that the utility of an RNA sample pooling strategy depends on the choice of the pooling parameters, such as the pool size and the number of RNA samples. Since the cost of RNA sample preparation is relatively low, one may consider using as many RNA samples as possible to capture the heterogeneity of the population under study, and using an adequate pooling strategy, one can substantially reduce the cost of the subsequent steps, which are considerably more expensive, and maintain the power of a DGE test. In particular, for scenarios with a high biological variability, a small pool size such as 2 can be effective to optimize the cost of the experiment and maintain the power that one would attain without pooling. Unlike the typical cost-saving strategies, such as reducing the sequencing depth or number of RNA samples (replicates), an adequate pooling strategy is particularly effective for scenarios with many genes with low and moderate levels of expression. We have demonstrated that pooling RNA samples or pooling RNA samples in conjunction with moderate reduction of the sequencing depth can be good options to further optimize the cost of the experiment without much loss of the power of the DGE test. The findings discussed in this paper can be useful for designing future experiments under possible constraints, such as limited budget, large biological variability, or insufficient RNA input.

## Methods

### RNA-seq datasets

Two publicly available bulk RNA-seq datasets were used in this study. The first is from Zhang et al. [18] (GEO accession number GSE49711), containing unstranded poly(A)+ RNA seq data from 498 neuroblastoma tumors. Paired-end sequencing (2 x 100 nucleotides) was done on a HiSeq 2000 instrument (Illumina). On average 20 million read pairs per sample were generated. Raw FASTQ files were processed with Kallisto v0.42.4 (index build with GRCh38-Ensembl v85). For this study, a subset of 172 patients with high-risk disease were selected, forming two groups: the MYCN amplified (*n*_1_ = 91) and MYCN non-amplified (*n*_2_ = 81) tumours. Further details about the Zhang data can be accessed in [18]. The second data set is from Assefa et al. [6] (GEO accession number GSE104756), containing stranded poly(A)+ RNA seq data from ten biological replicates of NGP neuroblastoma cells treated with either nutlin-3 or vehicle. Paired-end sequencing (2×75 nucleotides) was done on a NextSeq 500 instrument (Illumina). On average, 15 million read pairs per sample were generated. Raw FASTQ files were processed with Kallisto v0.42.4 (index build with GRCh38-Ensembl v85). The read quality assessment and validation was done using FASTQC, and subsequently quality metrics were aggregated using MultiQC [19] (v1.7) and are presented in Additional file 2.

### Differential gene expression analysis

For testing DGE, edgeR [4] and limma-voom [5] were used. These tools are commonly used tools for testing DGE, and implement different classes of models: edgeR fits negative binomial models on the read counts, whereas limma-voom fits normal linear models on the *log*_2_-counts per millions of reads. These tools also exhibit different performance with respect to their false-discovery rate control and sensitivity [6]. edgeR is implemented using *edgeR* [20] R Bioconductor package (v3.22.5) and limma-voom is implemented using *limma* [21] R Bioconductor package (v3.40.2).

### RNA-seq data simulation with built-in truth

We used the Zhang neuroblastoma RNA-seq data as source for the *SPsimSeq* [17] simulation. Upon first simulating the RNA-seq data for the reference scenario, subsequently, for each test scenario, data is generated from this reference scenario according to the design elements in Table 1a and using the data generation model in (1). The simulated datasets contain two groups of biological samples and 5000 genes of which 10% are DE between the groups (MYCN amplified vs MYCN not-amplified). Two series of simulations were run, with an absolute LFC estimate of the simulated DE genes of least 0.5 or 1, respectively. Afterwards, we calculate the actual FDR and TPR over 100 independent simulation runs for each particular simulation setting.

## Supplementary information

**Additional file 1** Supplementary result. This file constitutes of supplementary figures directly referred in this paper as well as the details of the data generating model for pooled experiments and power calculation.

**Additional file 2** multiQC report for NGP nutlin dataset. This file contains the quality metrics generated using the MultiQC tool for the NGP nutlin RNA-seq data.

## Data Availability

The RNA-seq data sets used in this study can be accessed from GEO repository with accession number GSE49711 (for Zhang data [18]) and GSE104756 (for NGP nutlin-3 data [6]). R software scripts used for generating the results presented in this paper can accessed at https://github.com/AlemuTA/RNA-sample-pooling-supplementary-results-.

## Abbreviations

cDNA: Complementary deoxyribonucleic acid
DE: Differential expression
DGE: Differential gene expression
FDR: False discovery rate
GEO: Gene expression omnibus
LFC: Log-fold-change
MAD: Mean absolute deviations
RNA: Ribonucleic acid
RNA-seq: RNA sequencing
TPR: True positive rate
