# Correction to: On the utility of RNA sample pooling to optimize cost and statistical power in RNA sequencing experiments

**DOI:** 10.1186/s12864-020-6754-2

**Published:** 2020-06-03

**Authors:** Alemu Takele Assefa, Jo Vandesompele, Olivier Thas

**Affiliations:** 1 Department of Data Analysis and Mathematical Modeling, Ghent University, 9000 Ghent, Belgium; 2 Department of Biomolecular Medicine, Ghent University, 9000 Ghent, Belgium; 3 Cancer Research Institute Ghent, Ghent University, Ghent, Belgium; 4 Center for Medical Genetics, Ghent University, Ghent, Belgium; 5 National Institute for Applied Statistics Research Australia (NIASRA), University of Wollongong, Wollongong, Australia; 6 Data Science Institute, I-BioStat, Hasselt University, Hasselt, Belgium

**Correction to: BMC Genomics 21, 312 (2020)**

**https://doi.org/10.1186/s12864-020-6721-y**

Following publication of the original article [1], an error was identified Eq. 3. In this Correction the erroneous and correct equation are given.

Equation 3 was previously given as:

$$ Var{Y}_k=\frac{2}{n\left(q+1\right)}\sum \limits_{j=1}^n\left({\mu}_j^2+{\sigma}_j^2\right)-\frac{1}{n^2}\sum \limits_{j=1}^n{\mu}_j^2+{\sigma}^2 $$

Equation 3 should be:

$$ Var\left\{{Y}_k\right\}=\frac{2}{n\left(q+1\right)}\sum \limits_{j=1}^n\left({\mu}_j^2+{\sigma}_j^2\right)-\frac{1}{n^2}\sum \limits_{j=1}^n{\mu}_j^2+{\sigma}^2 $$

The original article [1] has been corrected.
