# Supplementary material for: On the utility of RNA sample pooling to optimize cost and statistical power in RNA sequencing experiments
Source: BMC Genomics. 2020 Apr 19;21:312. doi: 10.1186/s12864-020-6721-y (PMC7168886; doi:10.1186/s12864-020-6721-y)
Supplement: Supplementary file 1 — Additional file 1 Supplementary result. This file constitutes of supplementary figures directly referred in this paper as well as the details of the data generating model for pooled experiments and power calculation. [file 12864_2020_6721_MOESM1_ESM.pdf]

Supplementary results: On the utility of RNA sample pooling to optimize cost and statistical power in RNA sequencing experiments

Alemu Takele Assefa, Jo Vandesompele, and Olivier Thas

October 28, 2019

Contents

1 Supplementary results 1: theoretical results **1**
1.1 Proofs for the mean and variance of Y_k 1
1.2 Estimation of the relative abundance and log-fold-change 4
1.3 Power calculation 6

2 Supplementary results 2: sample pooling results using the Zhang data **11**

3 Supplementary results 3: sample pooling results using the NGP nutlin data **16**

4 Supplementary results 4: additional results **18**

References **19**

1 Supplementary results 1: theoretical results

1.1 Proofs for the mean and variance of Y_k

We present here the proof for $\text{Var}\{Y_k\}$ in equation (3) in the manuscript. We first prove the expressions analytically and later we present the empirical confirmation using Monte-Carlo simulations. For a reminder, n is the number of biological samples, m is the number of pools, q is the number of biological samples per pool ($q = n/m$), U_j is the read counts of a given gene in biological sample $j = 1, 2, \ldots, n$, Y_k is the gene expression level in pool $k = 1, 2, \ldots, m$, W_{jk} denote the mixing weight for biological sample j in pool k, and A_{jk} is an indicator defined as 1 if biological sample j is in pool k, and 0 otherwise.

Proof. The data generating model in (1) is conditional on the pool size q, which is assumed to be fixed. This implies that A_{jk} is subject to the additional constraint $\sum_{j=1}^{n} A_{jk} = q$, which affects the variance calculation.

Without the constraint, if we let $Q = \sum_{j=1}^{n} A_{jk} \in \{0, 1, \ldots, n\}$, then $Q \sim \text{Binomial}(n, 1/m)$. Similarly, let $Q^{(j)} = \sum_{i \neq j} A_{ik} \in \{0, 1, \ldots, n-1\}$, then $Q^{(j)} \sim \text{Binomial}(n-1, 1/m)$. Therefore,

$$\text{P}(A_{jk} = 1 | Q = q) = \frac{\text{P}(Q = q | A_{jk} = 1)\text{P}(A_{jk} = 1)}{\text{P}(Q = q)} = \frac{\text{P}(Q^{(j)} = q - 1)\frac{1}{m}}{\text{P}(Q = q)}.$$

This is because $\text{P}(Q = q | A_{jk} = 1) = \text{P}(Q^{(j)} = q - 1)$. Therefore,

$$\text{P}(A_{jk} = 1 | Q = q) = \frac{\text{P}(Q^{(j)} = q - 1)\frac{1}{m}}{\text{P}(Q = q)} = \frac{\binom{\text{n-1}}{\text{q-1}} \left(\frac{1}{m}\right)^{q-1} \left(1 - \frac{1}{m}\right)^{n-q} \frac{1}{m}}{\binom{\text{n}}{\text{q}} \left(\frac{1}{m}\right)^{q} \left(1 - \frac{1}{m}\right)^{n-q}} = \frac{q}{n}.$$

Consequently,

$$\begin{aligned}
\mathrm{E}\left\{A_{jk}|Q=q\right\} &= \mathrm{P}(A_{jk}=1|Q=q) = \frac{q}{n},\\
\mathrm{Var}\left\{A_{jk}|Q=q\right\} &= (1-\mathrm{P}(A_{jk}=1|Q=q)) \times \mathrm{P}(A_{jk}=1|Q=q) = \frac{q(n-q)}{n^2}.
\end{aligned}$$

The same result can also be obtained if we translate the problem to an $m \times n$ contingency table with fixed row and column totals (similar constraints we have). That is, if pools ($k = 1, 2, \ldots, m$) are in the rows and the biological samples ($j = 1, 2, \ldots, n$) are in the columns, then all the row totals will be q and all the column totals will be 1, and A_{jk} are the $(ij)^{th}$ ellements of the table. This setting will give exactly the same mean and variance of A_{jk}.

In addition, under the assumption that the pooling weights $\boldsymbol{W}_k \sim \text{Dirichlet}(1, 1, \ldots, 1)$ in a given pool k, for the biological sample j (with $A_{jk} = 1$), $\mathrm{E}\left\{W_{jk}\right\} = 1/q$ and $\mathrm{Var}\left\{W_{jk}\right\} = \frac{q-1}{q^2(q+1)}$.

Therefore, based on these results

$$\begin{aligned}
\mathrm{E}\left\{Y_k\right\} &= \sum_{j=1}^{n} \mathrm{E}\left\{A_{jk}\right\} \mathrm{E}\left\{W_{jk}\right\} \mathrm{E}\left\{U_j\right\} + \mathrm{E}\left\{\varepsilon_k\right\}\\
&= \sum_{j=1}^{n} \frac{q}{n}\frac{1}{q}\mu_j = \frac{1}{n}\sum_{j=1}^{n}\mu_j.
\end{aligned}$$

For the variance, upon using the result that for n independent random variables $X_1, X_2, \ldots X_n$, $\mathrm{Var}\left\{\prod_{i=1}^{n} X_i\right\} = \prod_{i=1}^{n}\left(\mathrm{Var}\left\{X_i\right\} + \mathrm{E}\left\{X_i\right\}^2\right) - \prod_{i=1}^{n}\mathrm{E}\left\{X_i\right\}^2$, we find

$$\begin{aligned}
\mathrm{Var}\left\{Y_k\right\} &= \sum_{j=1}^{n} \mathrm{Var}\left\{A_{jk} \times W_{jk} \times U_j\right\} + \mathrm{Var}\left\{\varepsilon_k\right\}\\
&= \sum_{j=1}^{n}\Big[\Big\{\mathrm{Var}\left\{A_{jk}\right\} + \mathrm{E}\left\{A_{jk}\right\}^2\Big\}\Big\{\mathrm{Var}\left\{W_{jk}\right\} + \mathrm{E}\left\{W_{jk}\right\}^2\Big\}\Big\{\mathrm{Var}\left\{U_j\right\} + \mathrm{E}\left\{U_j\right\}^2\Big\} -\\
&\qquad \mathrm{E}\left\{A_{jk}\right\}^2 \mathrm{E}\left\{W_{jk}\right\}^2 \mathrm{E}\left\{U_j\right\}^2\Big] + \sigma^2\\
&= \sum_{j=1}^{n}\left[\left\{\frac{q(n-q)}{n^2} + \frac{q^2}{n^2}\right\}\left\{\frac{q-1}{q^2(q+1)} + \frac{1}{q^2}\right\}\left\{\sigma_j^2 + \mu_j^2\right\} - \frac{q^2}{n^2}\frac{1}{q^2}\mu_j^2\right] + \sigma^2\\
&= \frac{2}{n(q+1)}\sum_{j=1}^{n}(\sigma_j^2 + \mu_j^2) - \frac{1}{n^2}\sum_{j=1}^{n}\mu_j^2 + \sigma^2.
\end{aligned}$$

□

If $U_j \sim \text{Negative Binomial}(\mu_j, \phi)$, where $\mu_j = \rho L_j^0$, ρ is the relative abundance, L_j^0 is the library size in biological sample j (virtual library size), ϕ is the over-dispersion parameter, then, $\mathrm{Var}\left\{U_j\right\} = \sigma_j^2 = \mu_j + \phi\mu_j^2$. Therefore, the mean and variance of Y_k becomes,

$$\mathrm{E}\left\{Y_k\right\} = \frac{1}{n}\sum_{j=1}^{n}\mu_j \tag{1}$$

$$\mathrm{Var}\left\{Y_k\right\} = \frac{2}{n(q+1)}\sum_{j=1}^{n}(\mu_j + (\phi+1)\mu_j^2) - \frac{1}{n^2}\sum_{j=1}^{n}\mu_j^2 + \sigma^2. \tag{2}$$

To verify the mean and variance of Y_k (also the coefficient of variation) based on the expressions in (1) and (2), we set up a Monte-Carlo (MC) simulation with 2000 runs. In a given MC simulation $i,\ i = 1, 2, \dots, 2000$, generate $n = 60$ read counts from negative binomial distribution $U_j^{(i)} \sim \mathrm{NB}(\mu_j, \phi)$ and subsequently generates $Y_k^{(i)}$ using the data generating model (see equation (1) in the manuscript) for a pool size q. In a single MC simulation run i, the mean and variance of $Y_k^{(i)}$ are estimated by $\bar{Y}_i = m^{-1} \sum_{k=1}^{m} Y_k^{(i)}$ and $S_i^2 = (m-1)^{-1} \sum_{k=1}^{m} \left(Y_k^{(i)} - \bar{Y}_i\right)^2$, respectively. Afterwards, the $\mathrm{E}\{Y_k\} \approx 2000^{-1} \sum_{i=1}^{2000} \bar{Y}_i$ and $\mathrm{Var}\{Y_k\} \approx 2000^{-1} \sum_{i=1}^{2000} S_i^2$. Different choices of μ_j, ϕ, and q were considered. The results in Figure S1 show that the expressions in (1) and (2) are equivalent to the their corresponding MC approximations. This confirms that the expressions in (1) and (2) describe the true mean and variance of Y_k, respectively.

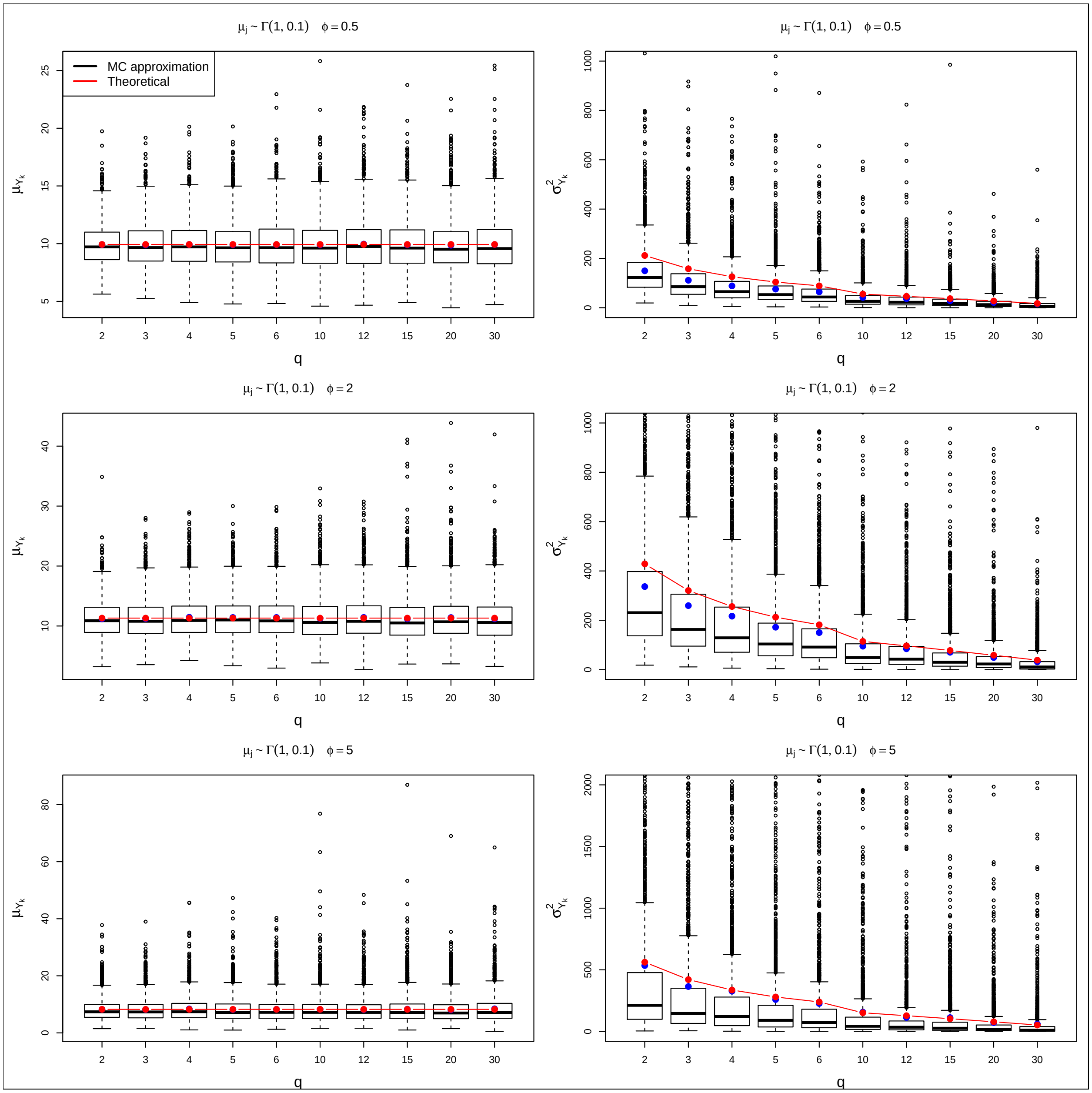

Figure S1: The Monte-Carlo and the analytical estimate of the mean (μ_Y) and variance of (σ_Y) of Y_k at different pool size (q). The solid red line indicates the analytical estimates, whereas the boxplots show the distribution of the the sample estimates in each Monte-Carlo simulation at each q. The Monte-Carlo estimates are the average across simulations in each q and they are indicated by solid black points on each boxplot.

1.2 Estimation of the relative abundance and log-fold-change

The moment estimator of the relative abundance ρ of a particular gene based on the gene expressions U_j from the n individual biological samples, is given by

$$\hat{\rho} = \frac{\sum_{j=1}^{n} U_j}{L_0}, \tag{3}$$

where $L_0 = \sum_{j=1}^{n} L_j^o$ is the total virtual library sizes, and L_j^o is the virtual library size in biological sample j. Similarly, we can drive the moment estimator of ρ based on gene expressions from the pooled samples $Y_k, k = 1, 2, \ldots, m$ starting from the sample mean of Y_k. That is, $\bar{Y} = \frac{1}{m}\sum_{k=1}^{m} Y_k \Rightarrow \mathrm{E}\left\{\bar{Y}\right\} = \frac{1}{n}\rho L_0$, and hence

$$\hat{\rho} = \frac{q}{L_0}\sum_{k=1}^{m} Y_k. \tag{4}$$

Note that, $\mathrm{E}\left\{\sum_{k=1}^{m} L_k\right\} = L_0/q$. Consequenty, $\hat{\rho}$ in equation (4) can be rewritten as $\hat{\rho} = \frac{\sum_{k=1}^{m} Y_k}{\mathrm{E}\{\sum_{k=1}^{m} L_k\}}$.

Now we compare the expectation and variance of $\hat{\rho}$ from the the standard experiment (3) and the pooled experiments (4). For this purpose, let $\hat{\rho}$ and $\hat{\rho}^*$ denote the estimates of the relative abundance from the standard and pooled experiment, respectively. Since we have used the moment estimators of ρ for both settings, it immediately follows that $\mathrm{E}\left\{\hat{\rho}\right\} = \mathrm{E}\left\{\hat{\rho}^*\right\} = \rho$.

It can be shown that

$$\mathrm{Var}\left\{\hat{\rho}\right\} = \frac{1}{L_0^2}\sum_{j=1}^{n} \sigma_j^2. \tag{5}$$

Using equation (3) of the main manuscript, it follows that

$$\begin{aligned}\mathrm{Var}\left\{\hat{\rho}^*\right\} &= \frac{q^2}{L_0^2}\sum_{k=1}^{m} \mathrm{Var}\left\{Y_k\right\} \\ &= \frac{2q}{q+1}\mathrm{Var}\left\{\hat{\rho}\right\} + \frac{2nq - q(q+1)}{n(q+1)L_0^2}\sum_{j=1}^{n} \mu_j^2 + \frac{nq}{L_0^2}\sigma^2.\end{aligned} \tag{6}$$

The right two terms of (6) are nearly 0 (division by very large number L_0^2) and have a negligible contribution to $\mathrm{Var}\left\{\hat{\rho}^*\right\}$. Consequently, we find

$$\frac{\mathrm{Var}\left\{\hat{\rho}^*\right\}}{\mathrm{Var}\left\{\hat{\rho}\right\}} \geq \frac{2q}{q+1}. \tag{7}$$

The expression in (7) implies that pooling leads to an estimate of the relative abundance which is at least $2q/(q+1)$ times more variable than the estimate we can obtain without pooling.

In DGE analysis, one essential statistic is the estimate of the biological effect (effect size). In many parametric methods, the log-fold-change (LFC) is commonly used to caliberate the biological effect size. As a result, we compare the LFC estimates from the standard and the pooled experiments. For testing DGE between two independent groups, the LFC of a particular gene is defined as $\theta = \log\frac{\rho_2}{\rho_1}$, where ρ_k is the relative abundance in group $k \in \{1, 2\}$. The estimate of θ for the standard experiment is given by $\hat{\theta} = \log\frac{\hat{\rho}_2}{\hat{\rho}_1}$ and for the pooled experiments $\hat{\theta}^* = \log\frac{\hat{\rho}_2^*}{\hat{\rho}_1^*}$.

Using the second-order Taylor expansion (the Delta method), we can approximate the variance of $\hat{\theta}$ and $\hat{\theta}^*$ as

$$\mathrm{Var}\left\{\hat{\theta}\right\} \approx \frac{\mathrm{Var}\left\{\hat{\rho}_2\right\}}{\rho_2^2} + \frac{\mathrm{Var}\left\{\hat{\rho}_1\right\}}{\rho_1^2}, \tag{8}$$

and

$$\mathrm{Var}\left\{\hat{\theta}^*\right\} \approx \frac{\mathrm{Var}\left\{\hat{\rho}_2^*\right\}}{\rho_2^2} + \frac{\mathrm{Var}\left\{\hat{\rho}_1^*\right\}}{\rho_1^2}. \tag{9}$$

Therefore, it follows that

$$\frac{\text{Var}\left\{\hat{\theta}^*\right\}}{\text{Var}\left\{\hat{\theta}\right\}} \geq \frac{2q}{q+1} \tag{10}$$

This also indicates that the LFC estimate from pooled experiments is at least $2q/(q+1)$ times more variable than that of the standard experiment. This is an important characteristic that affects the statistical power of a DGE test as shown in the next section.

1.3 Power calculation

Assume there is no pooling and we want to test for DGE between two independent groups of biological samples. Let U_{jk} denotes the read counts in biological sample $j = 1, 2, \ldots, n_k$ of group $k \in 1, 2$. Again we assume that $U_{jk} \sim$ Negative Binomial(μ_{jk}, ϕ), where ϕ is the over-dispersion parameter (assumed to be constant for all samples and all groups), and $\mu_{jk} = \text{E}\{U_{jk}\} = \rho_k L^0_{jk}$, where ρ_k is the relative abundance in group k and L^0_{jk} is the library size of biological sample j in group k. Let A_{jk} be the group label of U_{jk}, such that $A_{jk} = 0$ if $k = 1$ and $A_{jk} = 1$ if $k = 2$. n_k denotes the number of biological samples in group k, with $n = n_1 + n_2$. We want to test the null hypothesis $H_0 : \rho_1 = \rho_2$ against the alternative $H_A : \rho_1 \neq \rho_2$ at the α level of significance. In this section, we will calculate the statistical power of testing this hypothesis based on the method discussed in Zhu and Lakkis (2014).

We can fit the following negative binomial regression with L^0_{jk} as offset,

$$\log \mu_{jk} = \log\{\rho_k L^0_{jk}\} = \beta_0 + \beta_1 A_{jk} + \log L^0_{jk}, \tag{11}$$

where β_0 is the intercept and β_1 is the coefficient of the factor A. In this model, the parameter β_1 represents the LFC between the two groups, that is $\beta_1 = \log \frac{\rho_2}{\rho_1}$. This means, β_1 is equivalent to the LFC parameter introduced earlier as θ. Therefore, we can rewrite the hypothesis of DE as $H_0 : \beta_1 = 0$ against the alternative $H_A : \beta_1 \neq 0$.

If $\hat{\beta}_1$ is the maximum-likelihood estimator of β_1 (under H_A), then the variance of $\hat{\beta}_1$ is given by

$$\text{Var}\left\{\hat{\beta}_1\right\} = \frac{1}{n_1}\left[\frac{1}{\bar{L}^o}\left(\frac{1}{\rho_1} + \frac{1}{R\rho_2}\right) + \frac{(1+R)\phi}{R}\right] = \frac{1}{n_1}V_A, \tag{12}$$

where $R = n_2/n_1$, $\bar{L}^o = n^{-1}L_0$ (the mean library size across all biological samples). It is also easy to show that $\text{Var}\left\{\hat{\beta}_1\right\} = \text{Var}\left\{\hat{\theta}\right\}$, shown in (8). Under the null hypotsis,

$$\text{Var}\left\{\hat{\beta}_1\right\} = \frac{1}{n_1}\left[\frac{1}{\bar{L}^o}\left(\frac{1}{\tilde{\rho}_1} + \frac{1}{R\tilde{\rho}_2}\right) + \frac{(1+R)\phi}{R}\right] = \frac{1}{n_1}V_0, \tag{13}$$

where $\tilde{\rho}_1$ and $\tilde{\rho}_2$ are the true relative abundances under H_0, such that $\tilde{\rho}_1 = \tilde{\rho}_2 = \rho_1$.

Recall that our objective is to determine the power of testing the above hypothesis using the pooled experiment. Therefore, let $\hat{\beta}^*_1$ is the estimate of β_1 using the gene expression data from the pooled samples. $\hat{\beta}^*_1$ is the equivalent LFC in the pooled experiment, which was denoted by $\hat{\theta}^*$ earlier, i.e $\hat{\beta}^*_1 = \hat{\theta}^*$. In (10), we have established that $\text{Var}\left\{\hat{\theta}^*\right\} \geq \text{Var}\left\{\hat{\theta}\right\}\frac{2q}{q+1}$. Consequently, under the alternative hypothesis $\text{Var}\left\{\hat{\beta}^*_1\right\} \geq \frac{2q}{q+1}\text{Var}\left\{\hat{\beta}_1\right\}$ and under the null hypothesis $\text{Var}\left\{\hat{\beta}^*_1\right\}|_{H_0} \geq \frac{2q}{q+1}\text{Var}\left\{\hat{\beta}_1\right\}|_{H_0}$.

Therefore, given the pool size (q), the number of RNA samples in groups 1 and 2 (n_1 and n_2, respectively), the effect size to be detected θ, and over-dispersion ϕ, the power of the two-sided likelihood ratio test at significance level α can be calculated as,

$$\text{power} \leq \Phi\left(\frac{\sqrt{n_1(q+1)}|\theta| - Z_{\alpha/2}\sqrt{2qV_0}}{\sqrt{2qV_A}}\right), \tag{14}$$

where $\Phi(.)$ is the cumulative standard normal distribution, and $Z_{\alpha/2}$ is the $(1-\alpha/2)100\%$ quantile of the standard normal distribution. Note that in pooled experiments, n_1 and n_2 are the number of RNA samples before library prepartion.

In Figure S2 and S3, we present the relationship between the power and the total cost of data generation for different experimental design, including the sample pooling. In particular, we compare three cost-saving strategies and a reference scenario (the full budget experiment). These are

- *reference*: contains a total of n biological samples from two groups (each with $n/2$ samples) and there is no pooling. The average library size per sample is 20×10^6. The total cost is $C_t = C_{SP}\times n + C_{LP}\times n + C_S\times L_0$, where C_{SP}, C_{LP}, & C_S are sample preparation cost, library preparation cost and sequencing cost per 10^6, respectively.
- *Strategy A*: pooling experiment with pool size q. The $n/2$ RNA samples in each group are pooled to m pools $m = n/2q$ with average library size per pool is 20×10^6. Hence, the total cost is $C_t = C_{SP} \times n + C_{LP} \times 2m + C_S \times L_0/q$. This strategy reduces the library preparation and sequencing costs.
- *Strategy B*: reducing the number of biological samples (n) without pooling. Instead of the n total number of samples (in the reference design) we use n_s samples with $n_s/2$ per group with average library size per samples is 20×10^6. Hence, the total cost is $C_t = C_{SP} \times n_s + C_{LP} \times n_s + C_S \times (nL_0/n_s)$. This strategy reduces the sample preparation, library preparation and sequencing costs.
- *Strategy C*: reducing the sequencing depth. This is similar to the reference scenario, except that the average library size is reduced to L, where $L < 20 \times 10^6$. Hence, this strategy reduces only the sequencing cost by a factor l, $l = 20 \times 10^6/L$.

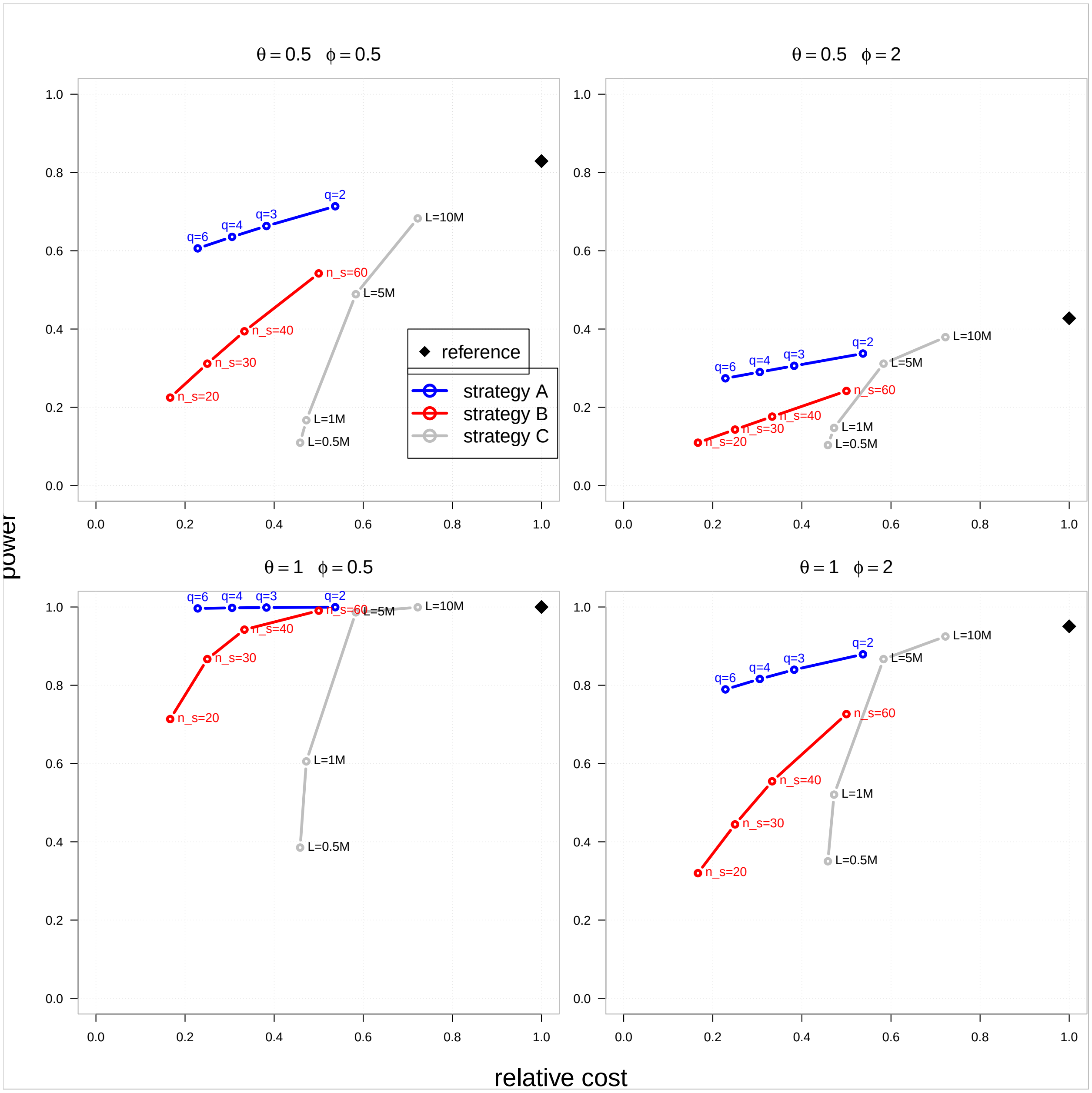

Figure S2: Zodiac plot representing power (at 5% significance level) versus the total cost of data generation. The gene expression levels are generated from $\mathrm{NB}(\rho L_j, \phi)$. This particular plot is for a gene with relative abundance of $\rho = 10^{-7}$ (low–abundance gene) in one of the groups. The reference strategy (denoted by a diamond shape) contains $n = 120$ biological samples (without pooling) with a mean library size of 20×10^6 per sample. Each panel represents a different LFC (θ) between the two groups and over-dispersion parameter ϕ (reflecting biological variability). The relative cost is determined as the total cost of each strategy divided by the total cost of the reference design.

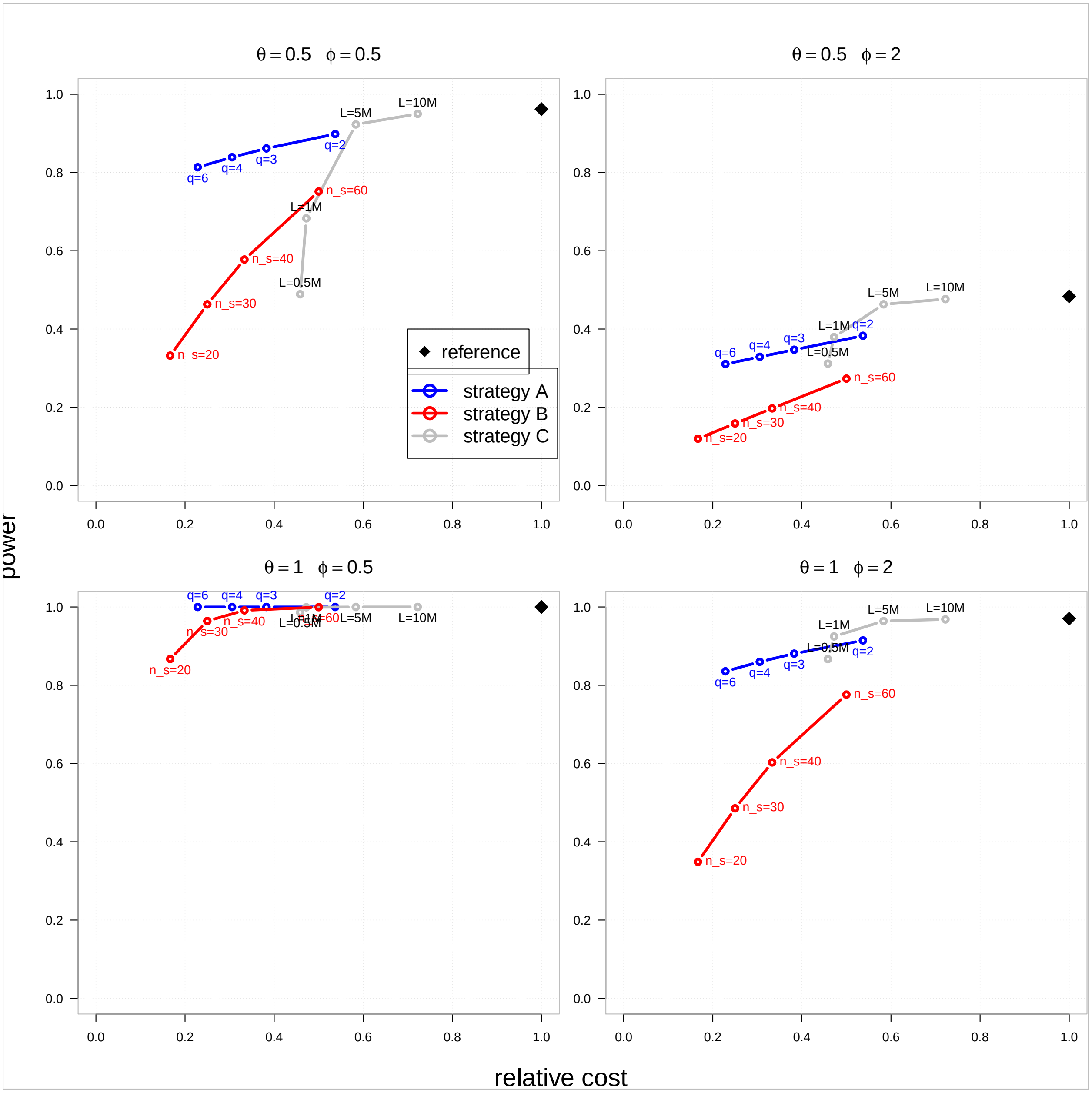

Figure S3: Zodiac plot representing power (at 5% significance level) versus the total cost of data generation. The gene expression levels are generated from $\mathrm{NB}(\rho L_j, \phi)$. This particular plot is for a gene with relative abundance of $\rho = 10^{-6}$ (moderate level of expression) in one of the groups. The reference strategy (denoted by a diamond shape) contains $n = 120$ biological samples (without pooling) with a mean library size of 20×10^6 per sample. Each panel represents a different LFC (θ) between the two groups and over-dispersion parameter ϕ (reflecting biological variability). The relative cost is determined as the total cost of each strategy divided by the total cost of the reference design.

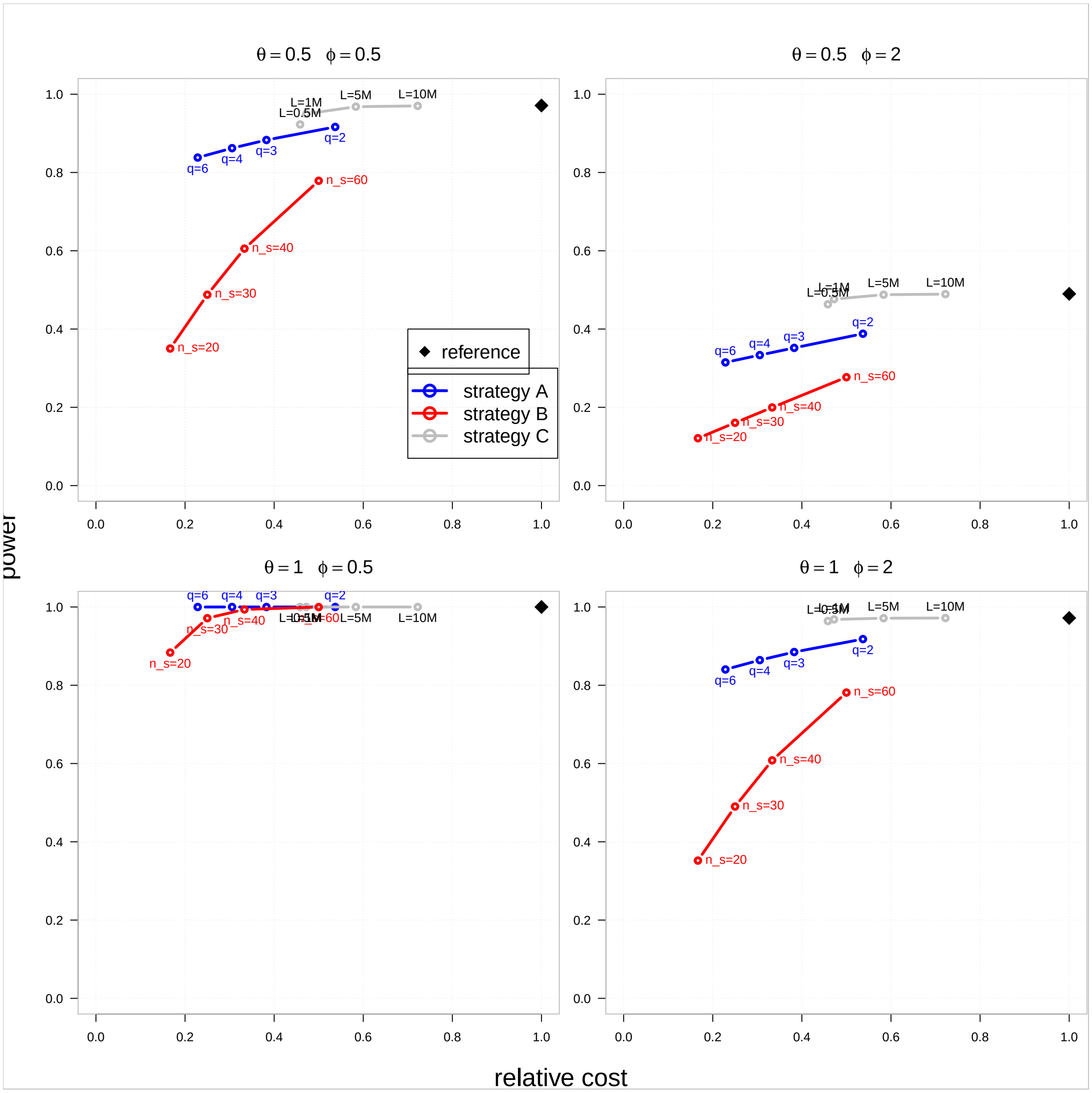

Figure S4: Zodiac plot representing power (at 5% significance level) versus the total cost of data generation. The gene expression levels are generated from $\mathrm{NB}(\rho L_j, \phi)$. This particular plot is for a gene with relative abundance of $\rho = 10^{-5}$ (high level of expression) in one of the groups. The reference strategy (denoted by a diamond shape) contains $n = 120$ biological samples (without pooling) with a mean library size of 20×10^6 per sample. Each panel represents a different LFC (θ) between the two groups and over-dispersion parameter ϕ (reflecting biological variability). The relative cost is determined as the total cost of each strategy divided by the total cost of the reference design.

2 Supplementary results 2: sample pooling results using the Zhang data

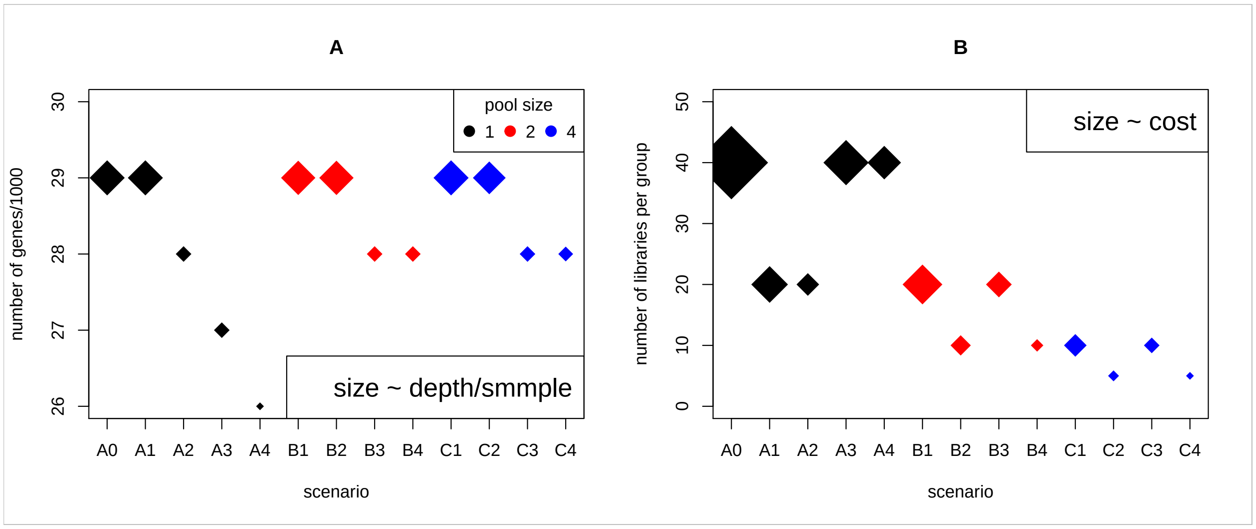

Figure S5: Sample level summaries. A) the number of genes with non-zero expressions in at least 3 libraries versus sequencing depth per library (symbol size); B) total cost (symbol size) versus number of libraries for each scenario.

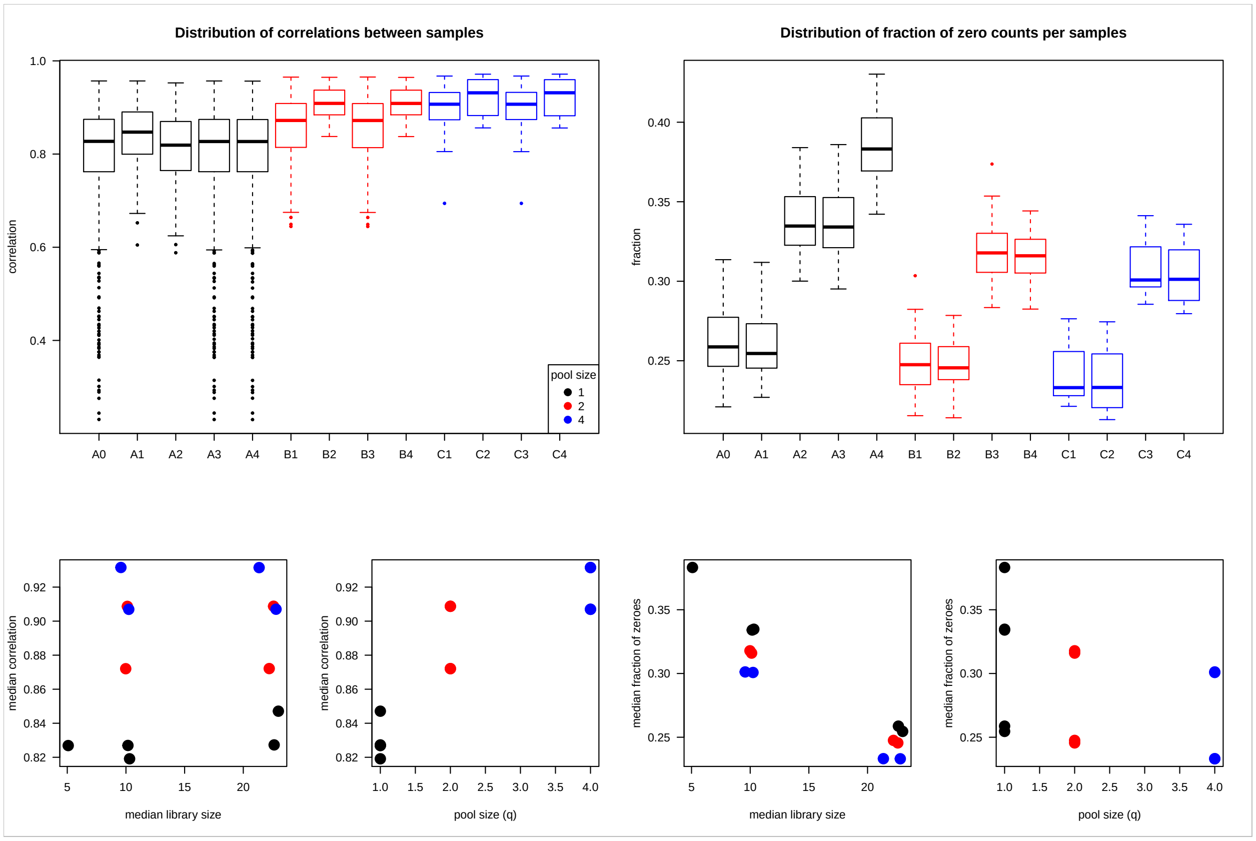

Figure S6: Sample level summaries of the observed data in each scenario in terms of (1) the distribution of the pairwise correlation coefficients between samples within a condition (MYCN status), and (2) the distribution of the fraction of zero counts observed in each sample. These summaries are also plotted as a function of the median library size and pool size in each scenario.

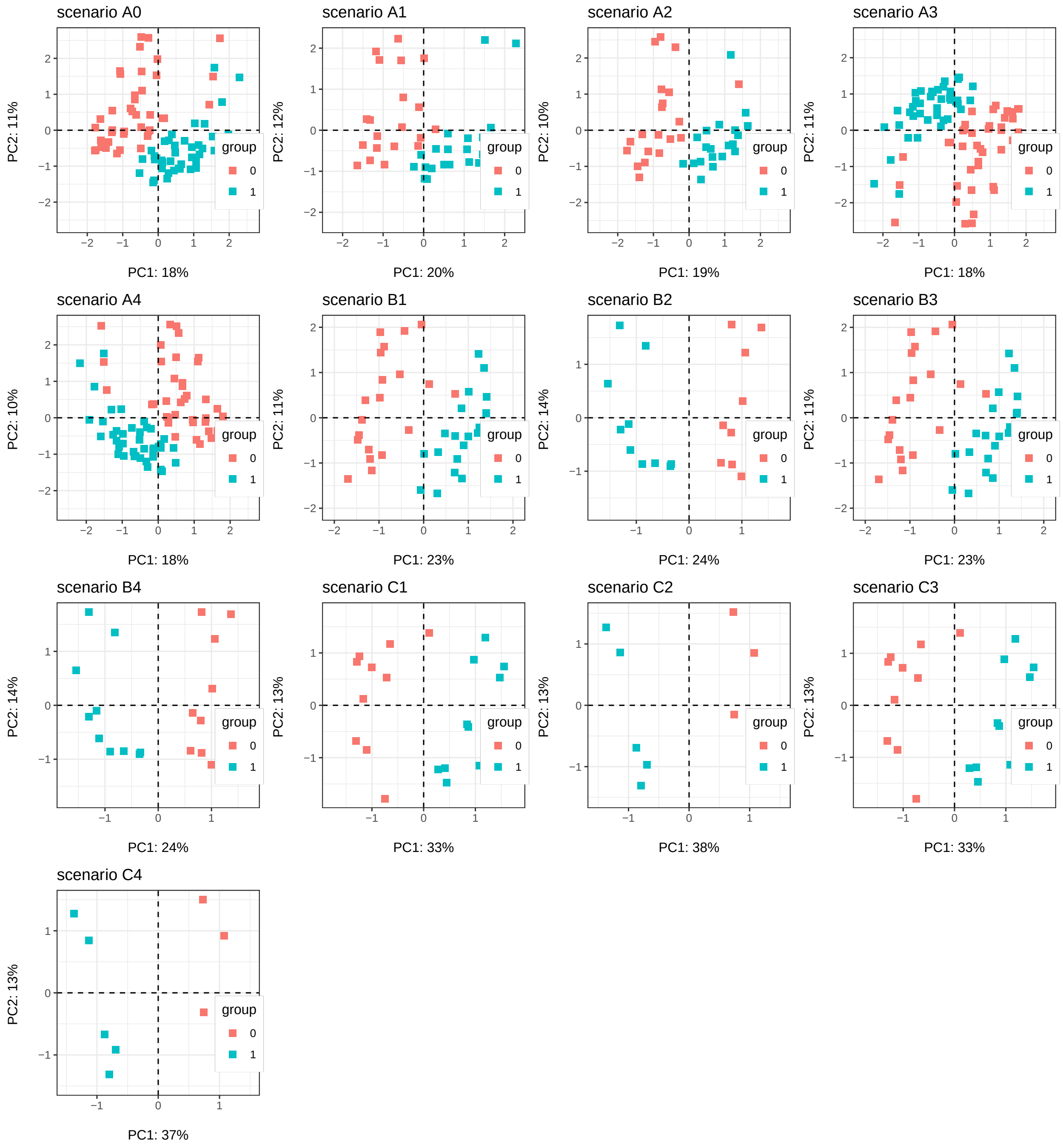

Figure S7: Two-dimensional visualization of neuroblastoma RNA samples (before and after pooling) using principal component analysis. The groups are defined as the MYCN status (group=1 for MYCN amplified and group=0 for MYCN non-amplified samples). In particular, the PCA was applied on the log-CPM transforemated read counts.

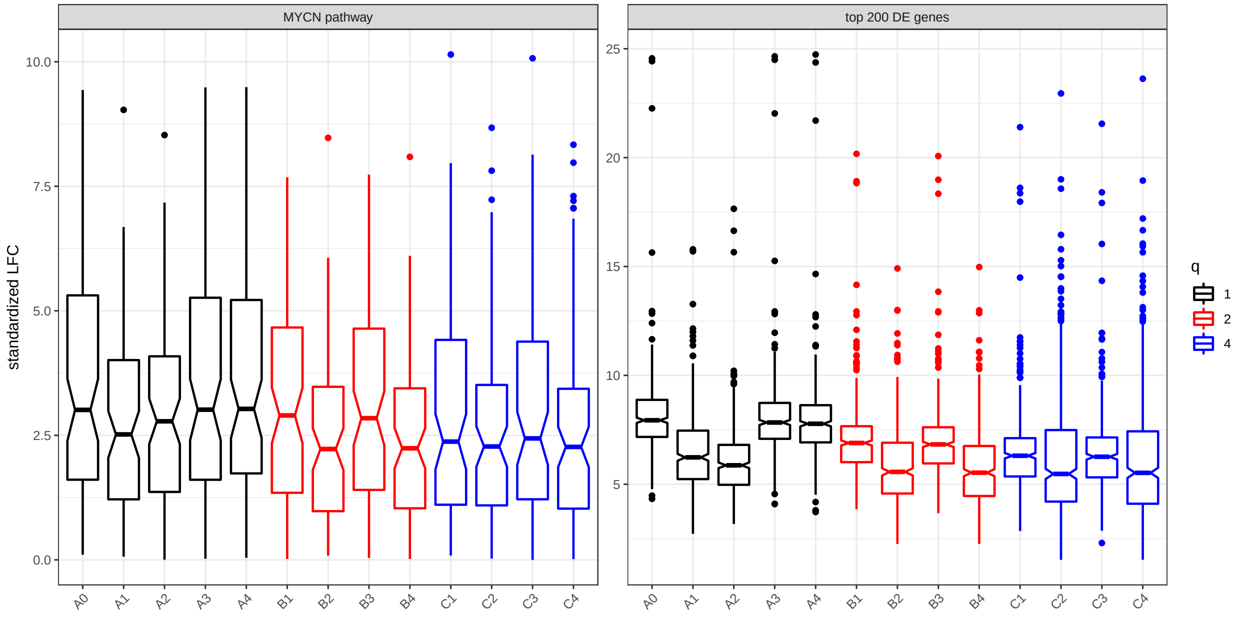

Figure S8: Standardized log-fold-change (LFC) for MYCN pathway genes and the top 200 DE genes detected in the reference scenario A0 using limma-voom

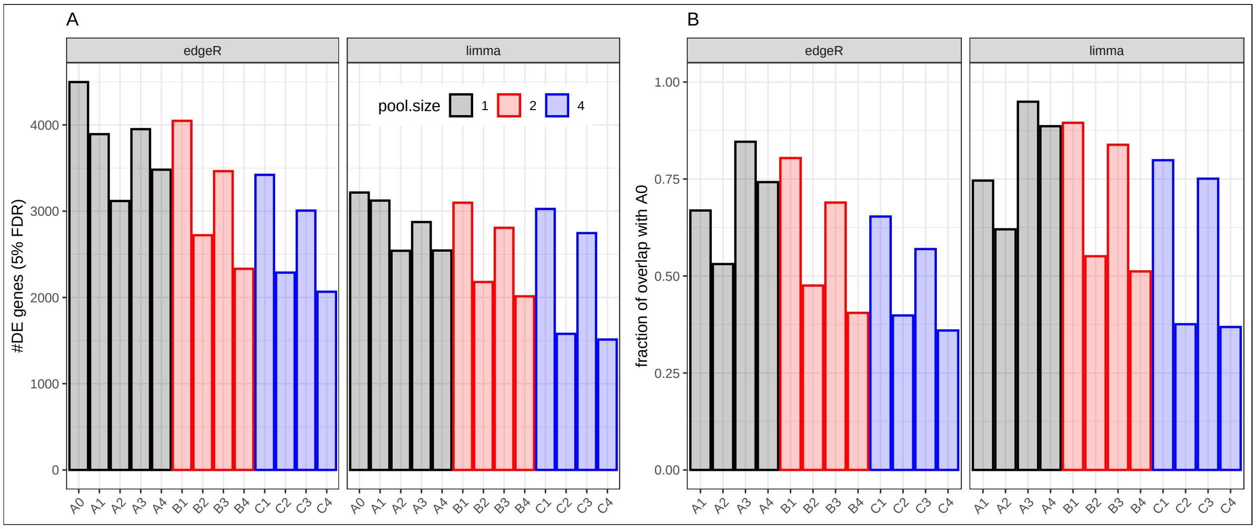

Figure S9: Differential gene expression results for pooling scenarios generated using the Zhang RNA-seq dataset. A) The number of DE genes detected at 5% FDR; B) The fraction of overlap (concordance) defined as the fraction of DE genes detected in a test scenario that are also detected in the reference scenario.

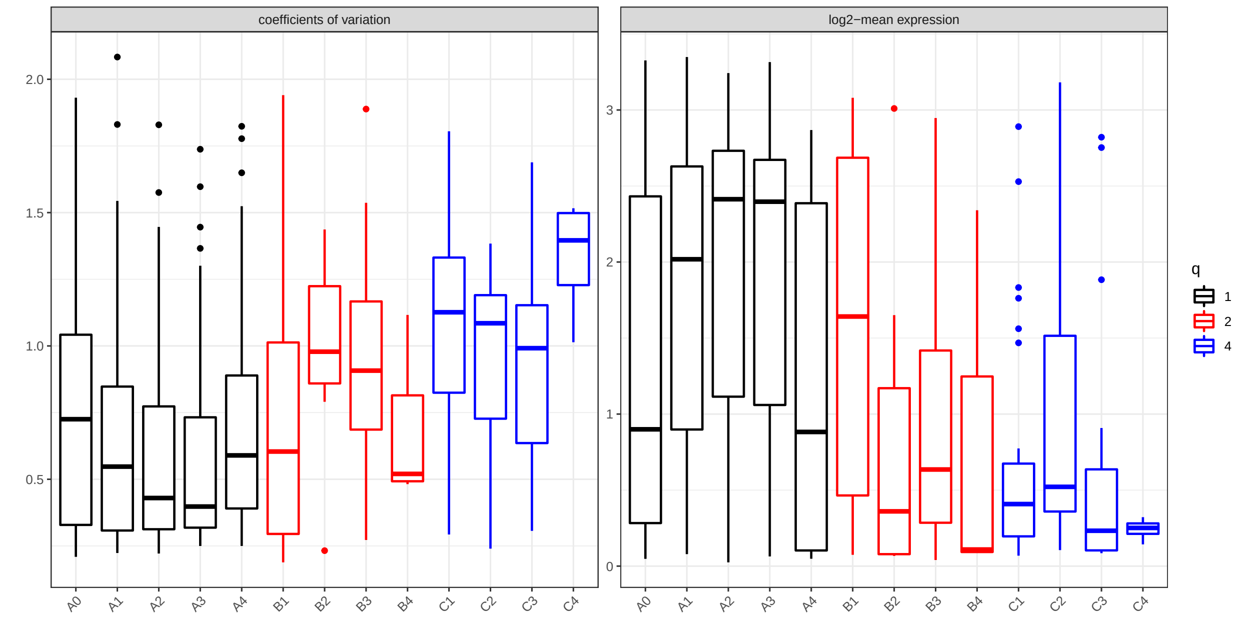

Figure S10: The distribution of the mean and coefficients of variation of the normalied read counts of the set of DE genes unquely detected by limma in each scenario.

3 Supplementary results 3: sample pooling results using the NGP nutlin data

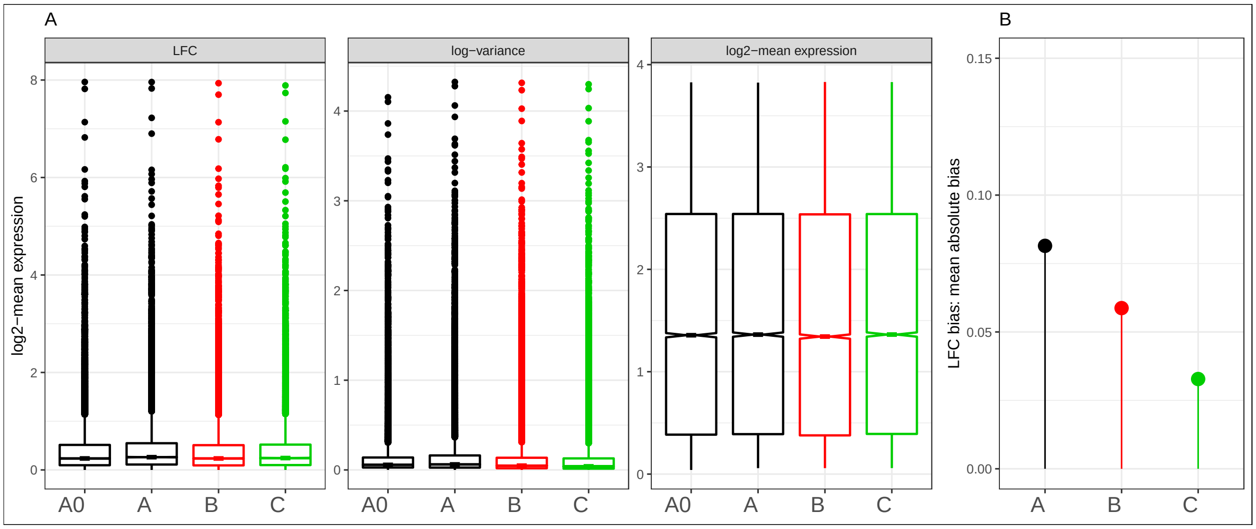

Figure S11: Summary of gene level characteristics for pooling scenarios generated using the NGP nutlin dataset. A) The distribution of $\log_2$-mean normalized expression of genes, the $\log_2$-vriance of normalized expression of genes, and the log-fold-change (LFC) between nutlin-3 and control; B) the estimated bias (mean absolute bias) of the three test scenarios relative to the reference scenario A0.

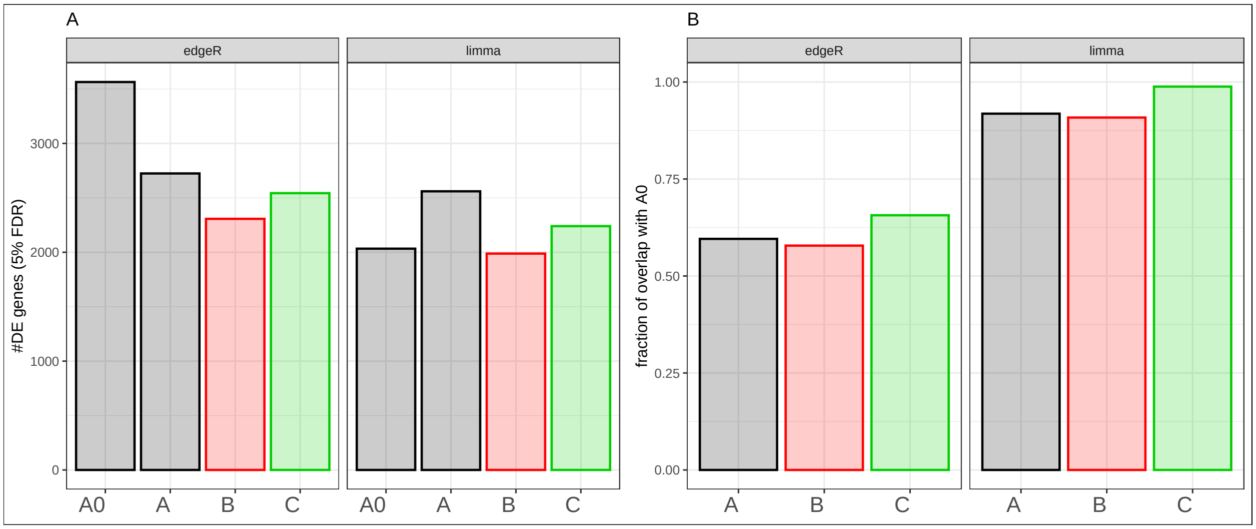

Figure S12: Differential gene expression results for pooling scenarios generated using the NGP nutlin RNA-seq dataset. A) The number of DE genes detected at 5% FDR; B) The fraction of overlap (concordance) defined as the fraction of DE genes detected in a test scenario that are also detected in the reference scenario.

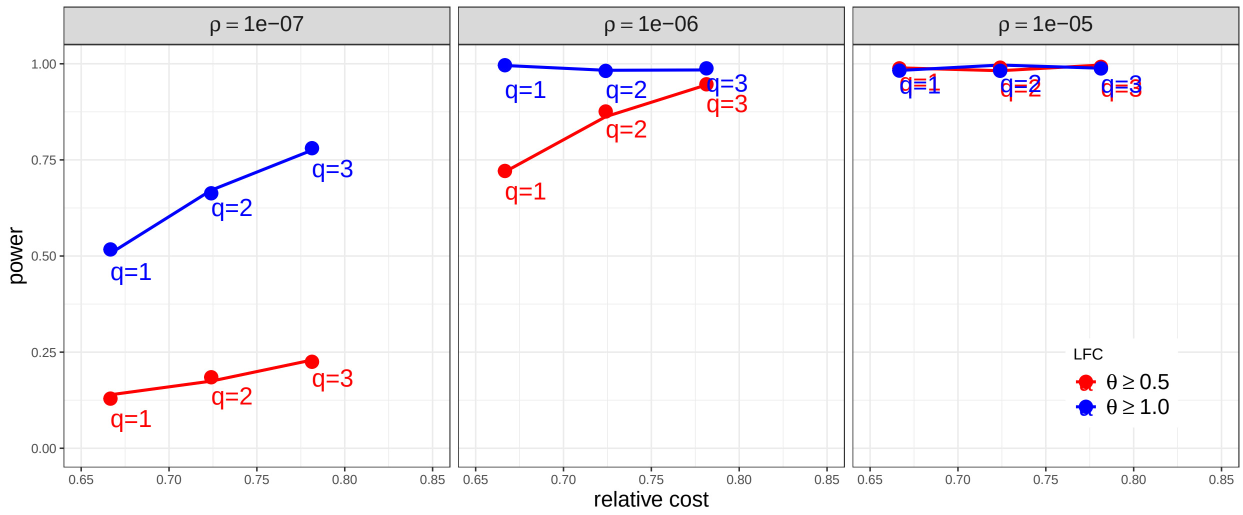

Figure S13: Zodiac plot representing power (at 5% significance level) versus the total cost of data generation for NGP cell line data. The gene expression levels are generated from NB$(\rho L_j, \phi)$, where ϕ is the common over-dispersion parameter for the NGP nutlin data (estimated using the edgeR package). The plots are generated for low, medium and high abundance genes with relative abundace of $\rho = 10^{-7}, \rho = 10^{-6}and\rho = 10^{-5}$, respectively. One unpooled design ($q = 1$) and two pooled designs ($q = 2$ and $q = 3$) were compared. These designs have equal number of replicates (3 replicates per group). That is, 3 individual cell lines ($q = 1$), 3 pools of 2 cell lines ($q = 2$) and 3 pools of 3 cell lines ($q = 3$). The mean library size per cell line is 15×10^6. The curves are generated for two different minimum LFCs (θ) between the two groups, $\theta \geq 0.5$ and $\theta \geq 1$. The relative cost is determined as the total cost of each strategy divided by the maximum total cost without pooling (9 cell lines per group).

4 Supplementary results 4: additional results

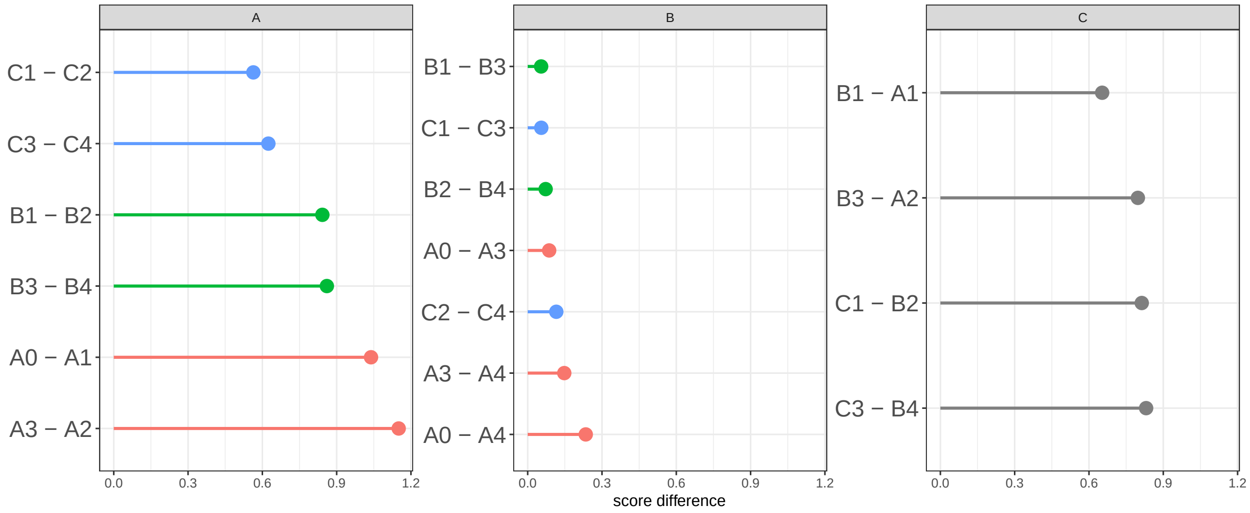

Figure S14: Pairwise comparison of scenarios based on the overall score for different characteristics. A) scenarios with different number of libraries but equal sequencing depth per library (demonstrating a sample size driven effect), B) scenarios with equal number of libraries but different sequencing depth per library (demonstrating a minor effect of sequencing depth), and C) scenarios with equal number of libraries and equal sequencing depth per library but different pool size (demonstrating a large pooling effect).

References

Zhu, Haiyuan, and Hassan Lakkis. 2014. "Sample Size Calculation for Comparing Two Negative Binomial Rates." *Statistics in Medicine* 33 (3): 376–87.
